# Ionic Recognition Controlled by Conformational Change: A DFT Investigation

**DOI:** 10.1021/acsomega.4c09597

**Published:** 2025-04-16

**Authors:** Renato P Orenha, Ana L. O. Andrade, Renato G. Rocha, Alvaro Muñoz−Castro, Thiago F. Santos, Maurício J. Piotrowski, Giovanni F. Caramori, Renato L. T. Parreira

**Affiliations:** † Núcleo de Pesquisas em Ciências Exatas e Tecnológicas, Universidade de Franca, Av. Dr. Armando Salles Oliveira 201, Franca, São Paulo 14404−600, Brazil; ‡ Facultad de Ingeniería, Arquitectura y Diseño, Universidad San Sebastián, Bellavista 7, Santiago 8420524, Chile; § Department of Physics, Federal University of Pelotas, Pelotas, Rio Grande do Sul 96010−900, Brazil; ∥ Departamento de Química, Universidade Federal de Santa Catarina, Campus Universitário Trindade, CP 476, Florianópolis, Santa Catarina 88040−900, Brazil

## Abstract

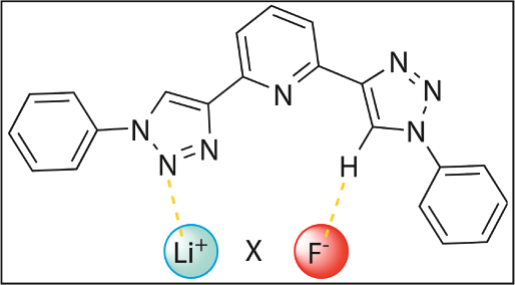

Ions play a crucial role in the production of important materials and are associated with various health and environmental issues. Noncovalent interactions serve as fundamental tools for controlling the availability of cations and/or anions. Herein, we investigate the ability of two conformations of the 2,6-bis(1,2,3-triazol-4-yl)pyridine molecule to recognize cations (**1**), such as Li^+^, Na^+^, or K^+^, and anions (**2**), including F^–^, Cl^–^, or Br^–^. EDA-NOCV analysis demonstrates that the conformers preferentially recognize ions based on the size of the cations (K^+^ → Na^+^ → Li^+^) and anions (Br^–^ → Cl^–^ → F^–^). The preferential interaction with smaller cations (and anions) arises from the more attractive electrostatic and orbital interactions (N^···.^cation and C–H^···.^anion bonds). The presence of electron-donor groups (−NH_2_) in the first conformer (**1**) enhances cation recognition through stronger electrostatic N^···.^cation interactions. Conversely, the presence of electron-acceptor groups (−NO_2_) in the second conformer (**2**) facilitates anion recognition via more favorable electrostatic, orbital, and dispersion C–H^···.^anion interactions. Cation recognition is found to be more favorable in the first conformer than anion recognition in the second due to more attractive electrostatic energy and/or less Pauli repulsive energy associated with (O or primarily N)^···.^cation interactions in **1**^···.^cations compared to (N or mainly C)–H^···.^anion bonds in **2**^···.^anions. These findings provide significant insights into the mechanisms of cation and/or anion recognition through different conformations using the same base structure and can inform the design of molecules with enhanced functionalities.

## Introduction

Ions play a crucial role in several important fields, including brick production,^1^ aluminum smelting,^2^ coal combustion,^3^ pesticide production,^4^ cement manufacturing,^5^ and the battery sector.^6^ Importantly, controlling the concentration of anions and cations is fundamental in reducing health-related issues, such as hormonal imbalances,^7^ impaired cognitive development,^8^ cardiovascular disease,^9,10^ and hypokalemia.^11^ Furthermore, monitoring ion levels in the environment is essential to avoid detrimental effects on aquatic ecosystems^12^ and to protect buildings and infrastructure from issues like acid rain.^13^

Traditionally, compounds have been designed to selectively recognize either anions^14^ or cations.^15^ However, recent developments have produced compounds capable of simultaneously interacting with both anions and cations, demonstrating a greater ability to recognize ions than their single-ion counterparts.^16,17^ More complex structures can feature multiple ion-binding sites with varying affinity levels, allowing for more selective ionic recognition.^18^ Another class of molecules, known as molecular tweezers, consists of a spacer structure bridging two interacting sites, often facilitated by conformational changes,^19^ which can recognize either anions^20^ or cations.^21^

**Figure 1:**
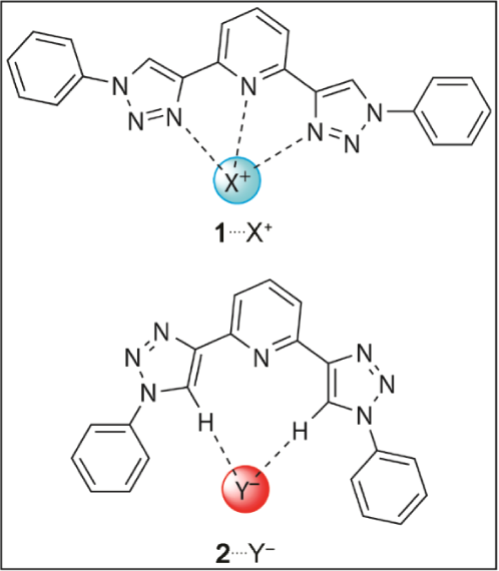
Recognition of cations (X^+^) and anions (Y^–^) supported by the **1** and **2** conformers, respectively.

In particular, the [2,6-bis(1,2,3-triazol-4-yl)pyridine] (**btp**) derivative is capable of recognizing both cations and anions using two distinct conformations: **1** and **2** (Figure 1).^22^ X-ray crystallography results indicate that the more stable conformer of the isolated **btp** molecule has its nitrogen atoms in the triazole rings pointing away from the pyridyl nitrogen.^23^ Additionally, single-crystal X-ray diffraction data suggest that one conformer can recognize cations via nitrogen atoms, while the other can interact with anions through C–H groups.^24^

**Figure 2:**
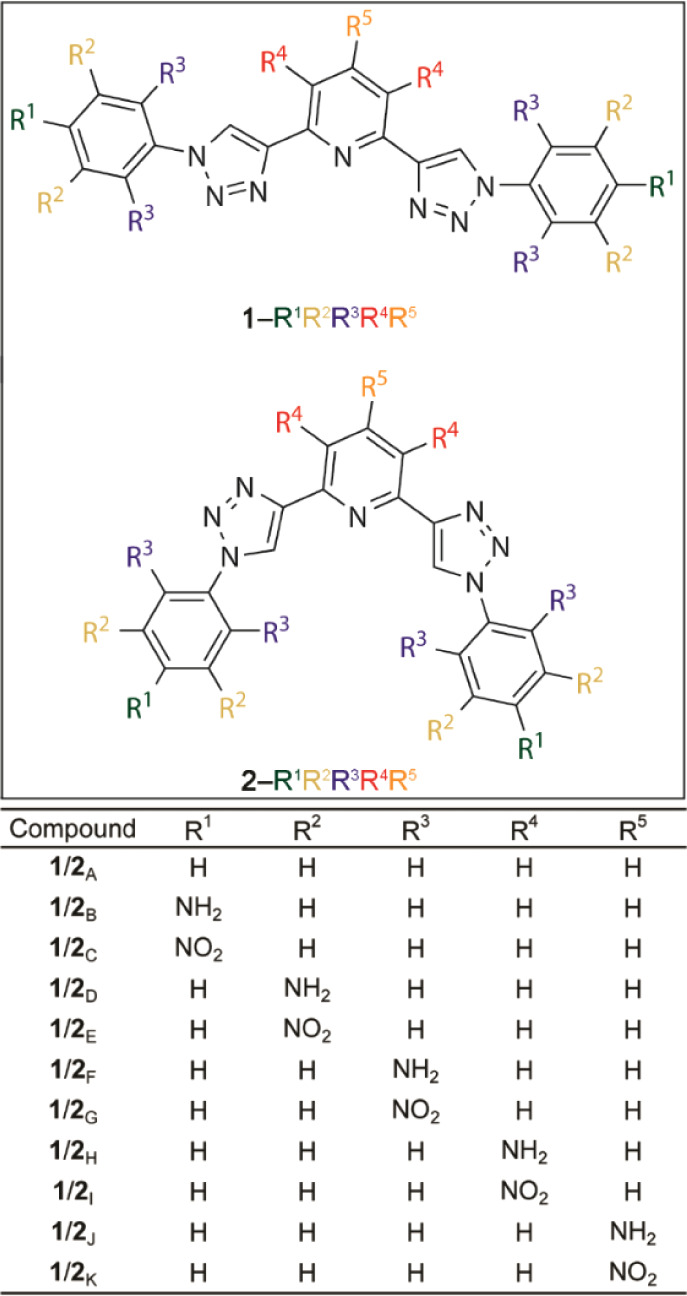
Structures of the substituted conformers: **1**_A–K_ and **2**_A–K_.

This study investigates the ability of the **1** and **2** conformers to interact with cations (Li^+^, Na^+^, and K^+^) and anions (F^–^, Cl^–^, and Br^–^) of different natures. Structural modifications (replacing −H atoms with electron-donating −NH_2_ or electron-withdrawing −NO_2_ groups) are proposed to tune the chemical interactions of **1**^···.^cations or **2**^···.^anions (Figure 2). The analysis focuses on the preferential chemical interaction between **1**^···.^cations or **2**^···.^anions. The bonding situations are investigated using energy decomposition analysis (EDA) in conjunction with the natural orbitals for chemical valence (NOCV) methodology. Main electrostatic interactions are evaluated using electrostatic potential (ESP) surfaces, while a topological analysis of the electron density is performed using the quantum theory of atoms in a molecule (QTAIM) method.

## Results and Discussion

### Cationic Recognition

EDA was employed to elucidate the bonding mechanism between, for example, conformer **1**_**A**_ and Na^+^. The interaction energy, Δ*E*_int_, is composed of four main components:^25^
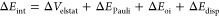
1

The electrostatic energy, Δ*V*_elstat_, accounts for classical electrostatic interactions between the non-perturbed charge distributions of the interacting fragments. The Pauli repulsion term, Δ*E*_Pauli_, includes destabilizing interactions between occupied orbitals, which are related to steric effects. The orbital interaction energetic term, Δ*E*_oi_, considers charge transfer (the interaction between occupied orbitals in one fragment and unoccupied orbitals in another) and polarization (the mixing of occupied and unoccupied orbitals due to the presence of another fragment). The Δ*E*_disp_ component also accounts for dispersion corrections, as proposed by Grimme et al.^26,27^

The EDA results for the interactions between the **1**_A–K_ conformers and cations (Li^+^, Na^+^, or K^+^) are summarized in Table 1. For the interactions involving **1**_A–K_^···.^(Li^+^, Na^+^, or K^+^), the contributions from Δ*V*_elstat_ (63–70%) and Δ*E*_oi_ (26–34%) are comparable, while Δ*E*_disp_ contributes a smaller portion (3–6%) to the total stabilization energy, Δ*V*_elstat_ + Δ*E*_oi_ + Δ*E*_disp_. These findings indicate that the bonds possess a predominantly noncovalent or polar covalent nature.

**Table 1:** Analysis of the Bonding Situations between the Receptors (**1**_**A**–**K**_) and Cations (Li^+^, Na^+^, or K^+^) Using the EDA-NOCV Methodology^a^^b^,^c^

Complex	Δ*E*_int_	Δ*V*_elstat_	Δ*E*_Pauli_	Δ*E*_oi_	Δ*E*_disp_	Δ*E*_oi,1_	Δ*E*_oi,2_	Δ*E*_oi,3_
**1**_**A**_^···.^Li^+^	–108.07	–76.80 (63)	14.77	–42.03 (34)	–4.01 (3)	–8.24	–7.86	–4.01
**1**_**A**_^···.^Na^+^	–85.94	–72.40 (68)	20.07	–28.80 (27)	–4.80 (5)	–4.83	–3.15	–3.29
**1**_**A**_^···.^K^+^	–65.81	–64.49 (69)	27.49	–24.19 (26)	–4.61 (5)	–4.48	–2.94	–2.84
**1**_**B**_^···.^Na^+^	–92.64	–81.01 (70)	23.07	–29.99 (26)	–4.72 (4)	–4.17	–3.36	–4.07
**1**_**C**_^···.^Na^+^	–76.69	–63.93 (65)	21.68	–29.71 (30)	–4.74 (5)	–4.39	–3.64	–3.77
**1**_**D**_^···.^Na^+^	–92.18	–80.67 (70)	22.84	–29.62 (26)	–4.73 (4)	–4.81	–3.32	–3.03
**1**_**E**_^···.^Na^+^	–72.40	–59.32 (63)	21.16	–29.49 (32)	–4.75 (5)	–4.66	–3.53	–3.26
**1**_**F**_^···.^Na^+^	–85.91	–74.47 (69)	22.74	–29.40 (27)	–4.78 (4)	–4.90	–3.25	–2.82
**1**_**G**_^···.^Na^+^	–84.48	–64.13 (63)	17.66	–31.91 (31)	–6.11 (6)	–5.24	–4.16	–3.37
**1**_**H**_^···.^Na^+^	–90.61	–79.50 (69)	23.96	–30.36 (26)	–4.72 (4)	–4.56	–4.38	–3.10
**1**_**I**_^···.^Na^+^	–75.76	–63.85 (65)	22.98	–30.01 (30)	–4.88 (5)	–4.80	–3.75	–3.24
**1**_**J**_^···.^Na^+^	–91.12	–79.63 (70)	23.14	–29.92 (26)	–4.71 (4)	–4.80	–3.30	–3.42
**1**_**K**_^···.^Na^+^	–79.94	–67.28 (66)	21.27	–29.22 (29)	–4.72 (5)	–4.78	–3.12	–3.23

a The energy unit is kcal mol^–1^.

b Δ*E*_int_ = Δ*V*_elstat_ + Δ*E*_Pauli_ + Δ*E*_oi_ + Δ*E*_disp_.

c Values in parentheses represent the percentage of each stabilizing contribution (Δ*V*_elstat_ + Δ*E*_oi_ + Δ*E*_disp_ = 100%).

Initially, the ability of conformer **1** to recognize cations of varying sizes is examined through the analysis of **1**_A_^···.^(Li^+^, Na^+^, or K^+^) interactions. The results reveal a more favorable Δ*E*_int_ energy as the size of the cation decreases. This trend can be rationalized by the stronger attractive contributions from Δ*V*_elstat_ and Δ*E*_oi_ and less repulsive Δ*E*_Pauli_ values in the **1**_A_^···.^Li^+^ interaction compared to **1**_A_^···.^Na^+^ and **1**_A_^···.^K^+^. The Δ*E*_disp_ energy remains similar across the **1**_A_^···.^(Li^+^, Na^+^, or K^+^) interactions. Further investigation into the trends associated with Δ*V*_elstat_ will be conducted using the ESP surfaces. Additionally, the influence of Δ*E*_oi_ will be explored using NOCV and QTAIM methodologies.

**Figure 3:**
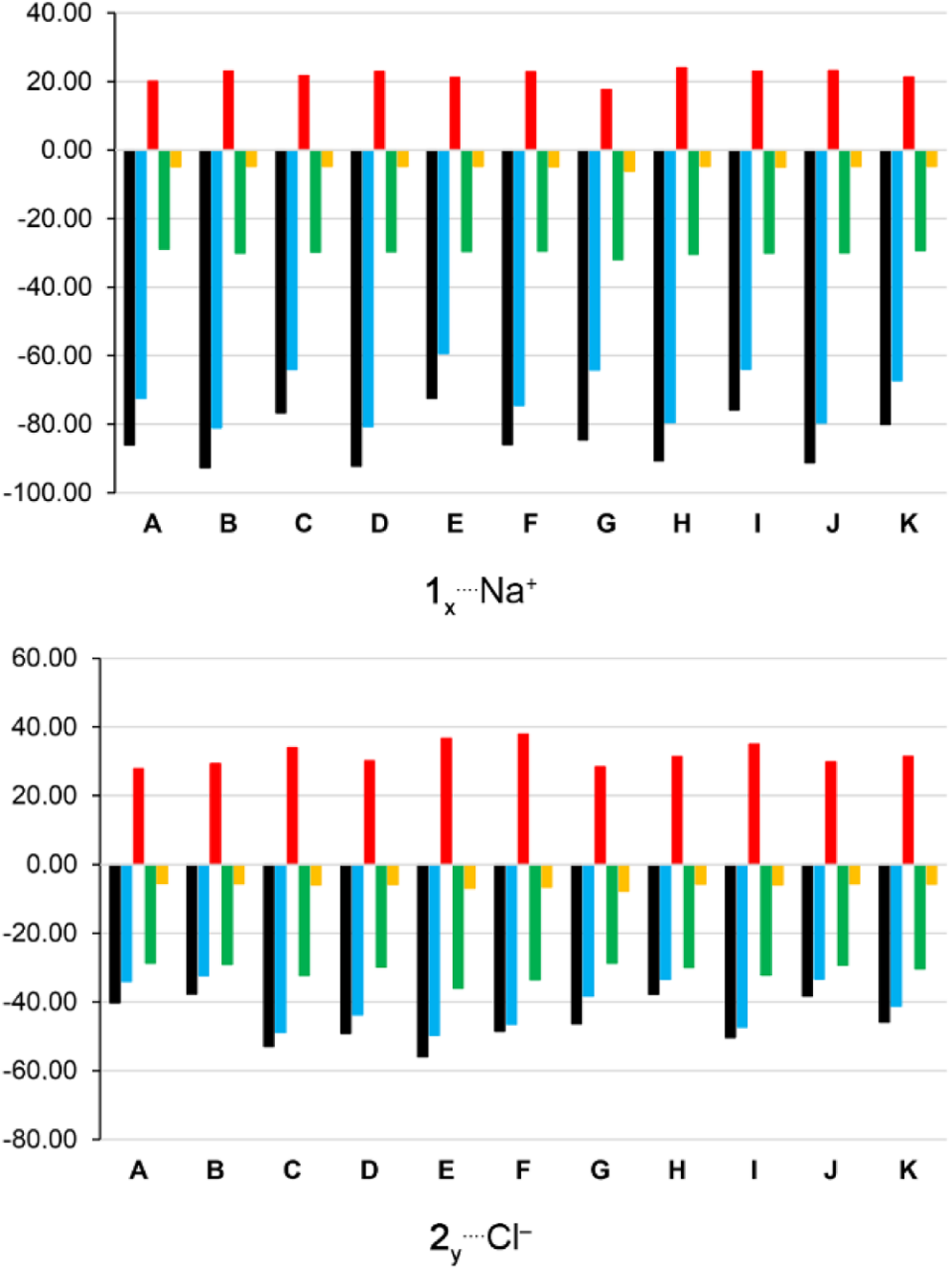
EDA results for the **1**_*x*_^···.^Na^+^ and **2**_*y*_^···.^Cl^–^ structures (where _*x*_ or _*y*_ = **_A_**_–**K**_). Bar color codes are as follows: Δ*E*_int_ = black; Δ*V*_elstat_ = blue; Δ*E*_Pauli_ = red; Δ*E*_oi_ = green; and Δ*E*_disp_ = yellow.

The importance of electron-donating (−NH_2_) or electron-withdrawing (−NO_2_) groups at different positions for Na^+^ recognition was studied using the **1**_A–K_ series (Figure 2). Overall, substitution of −H by −NH_2_ in **1**_**A**_ enhances the recognition of Na^+^, resulting in more attractive **1**_(**B**, **D**, **H,** or **J**)_^···.^Na^+^ interactions compared to the **1**_**A**_^···.^Na^+^ bond (Figure 3). This is attributed to the more favorable Δ*E*_oi_ and, mainly, Δ*V*_elstat_ energies in the **1**_(**B**, **D**, **H,** or **J**)_^···.^Na^+^ interactions compared to **1**_**A**_^···.^Na^+^. The **1**_**F**_ receptor shows similar interaction energy with the Na^+^ ion compared to the **1**_**A**_ receptor. This occurs because the more attractive Δ*V*_elstat_ energetic term is counterbalanced by the more repulsive Δ*E*_Pauli_ energy component in the **1**_**F**_^···.^Na^+^ complex compared to the **1**_**A**_^···.^Na^+^ structure. In general, the Δ*E*_disp_ values are similar across the **1**_(**A**, **B**, **D**, **F**, **H,** or **J**)_^···.^Na^+^ molecules.

Conversely, the presence of electron-withdrawing (−NO_2_) groups in the **1**_**A**_ conformer hampers Na^+^ recognition. The **1**_(**C**, **E**, **G**, **I,** or **K**)_^···.^Na^+^ bonds exhibit less attractive characteristics compared to the reference **1**_**A**_^···.^Na^+^ interaction (Figures 2 and 3). This is due to the less favorable Δ*V*_elstat_ energy and the more repulsive Δ*E*_Pauli_ values in the **1**_(**C**, **E**, **I,** or **K**)_^···.^Na^+^ interactions relative to the **1**_**A**_^···.^Na^+^ bond. Overall, the Δ*E*_oi_, and Δ*E*_disp_ values are comparable in the **1**_(**A**, **C**, **E**, **I,** or **K**)_^···.^Na^+^ compounds. Exceptionally, there is a more favorable Δ*E*_oi_ energy in the **1**_**I**_^···.^Na^+^ bond compared to **1**_**A**_^···.^Na^+^ interaction. Notably, the **1**_**G**_ molecule shows close interaction energy with respect to Na^+^ recognition when compared to the **1**_**A**_ compound (Figure 2). This can be explained by the more favorable Δ*E*_oi_ and Δ*E*_disp_ energies and by the less repulsive Δ*E*_Pauli_ value present in the **1**_**G**_^···.^Na^+^ bond with respect to the **1**_**A**_^···.^Na^+^ interaction (Figure 3).

**Figure 4:**
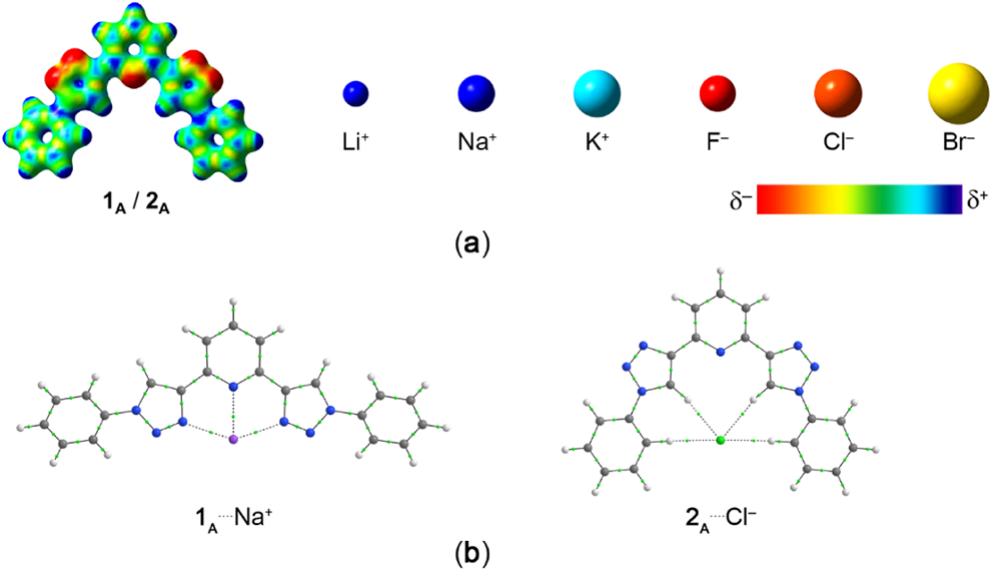
(**a**) Electrostatic potential surfaces mapped onto electronic densities of (i) 0.050 au [ranging from 0.100 au (red) to 0.250 au (blue)] for the **1**_**A**_/**2**_**A**_ receptors and (ii) 0.020 au [ranging from 0.000 au (red) to 0.600 au (blue)] for Li^+^, Na^+^, and K^+^ cations and [ranging from – 0.300 au (red) to 0.000 au (blue)] for Cl^–^, Br^–^, and I^–^ anions. (**b**) Topological map showing bond paths (continuous or dashed lines connecting the cores) and bond critical points (small light green points) for the **1**_**A**_^···.^Na^+^ and **2**_**A**_^···.^Cl^–^ complexes. Color code for atoms: H = white, C = gray, N = blue, Na = purple, and Cl = green.

To elucidate the trends in Δ*V*_elstat_, ESP surfaces of the isolated compounds **1**_**A**–**K**_ and the cations (Li^+^, Na^+^, and K^+^) were calculated (Figures 4a and S1). In these surfaces, red regions indicate high electron concentration (e.g., around nitrogen or oxygen atoms), while blue regions, close to hydrogen atoms, signify low electron density.^28^ The high electron density in the nitrogen or oxygen atoms of the isolated **1**_**A**–**K**_ structures suggests their suitability for interaction with the low electron concentration regions in the isolated cations (Li^+^, Na^+^, or K^+^). For a more quantitative analysis of the electrostatic interactions between **1**_**A**–**K**_ and cations (Li^+^, Na^+^, or K^+^), the minimum and maximum ESP values for the N/O atoms and alkali metal cations, corresponding to (N or O)^···.^(Li^+^, Na^+^, or K^+^) bonds, are organized in Table 2. The increase in the maximum ESP value with the decrease in cation size accounts for the more attractive Δ*V*_elstat_ energy in the **1**_A_^···.^Li^+^ bond compared to the **1**_A_^···.^Na^+^ and **1**_A_^···.^K^+^ interactions.

**Table 2:** Selected ESP Values (kcal mol^–1^) for the Isolated Structures

Structure	ESP_N_^a^	ESP_O_^NO2^^b^	ESP_H_^c^	ESP_H_^NH2^^d^	ESP_C_^NO2^^e^	ESP_Cation_^f^	ESP_Anion_^g^
**1**_**A**_/**2**_**A**_	–27.48	–	30.44	–	–	–	–
**1**_**B**_/**2**_**B**_	–32.20	–	24.91	–	–	–	–
**1**_**C**_/**2**_**C**_	–16.86	–	45.96	–	–	–	–
**1**_**D**_/**2**_**D**_	–31.92	–	24.64	38.61	–	–	–
**1**_**E**_/**2**_**E**_	–14.32	–	45.82	–	–	–	–
**1**_**F**_/**2**_**F**_	–27.77	–	18.43	34.20	–	–	–
**1**_**G**_/**2**_**G**_	–25.91	–24.91	23.75	–	24.23	–	–
**1**_**H**_/**2**_**H**_	–27.29	–	26.97	–	–	–	–
**1**_**I**_/**2**_**I**_	–21.69	–	38.94	–	–	–	–
**1**_**J**_/**2**_**J**_	–29.66	–	27.72	–	–	–	–
**1**_**K**_/**2**_**K**_	–23.00	–	36.02	–	–	–	–
Li^+^	–	–	–	–	–	339.38	–
Na^+^	–	–	–	–	–	248.90	–
K^+^	–	–	–	–	–	190.58	–
F^–^	–	–	–	–	–	–	–180.11
Cl^–^	–	–	–	–	–	–	–145.72
Br^–^	–	–	–	–	–	–	–134.69

a Average of the minimum ESP values present in the nitrogen atoms associated with the N^···.^cation interactions.

b Average of the minimum ESP values present in the oxygen atoms correlated to the ONO^···.^Na^+^ interaction.

c Average of the maximum ESP values located in the hydrogen atoms involved in the C–H^···.^anion interactions.

d Average of the maximum ESP values located in the hydrogen atoms of the −NH_2_ group related to the HN–H^···.^anion interaction.

e Maximum ESP value located in the carbon atom of the C–NO_2_ group associated with the O_2_N–C^···.^anion interaction.

f Maximum ESP value located in the isolated cation.

g Minimum ESP value located in the isolated anion.

The substitution of −H with −NH_2_ groups at positions −R^1–5^ in **1**_A_ to form **1**_(**B**, **D**, **F**, **H,** or **J**, respectively)_ compounds results in a similar or decreased minimum ESP value for the nitrogen atoms associated with the N^···.^Na^+^ interactions (Table 2). These results explain the enhanced Δ*V*_elstat_ energy in the **1**_(**B**, **D**, **F**, **H,** or **J**)_^···.^Na^+^ interactions compared to the **1**_**A**_^···.^Na^+^ bond (Table 1).

The presence of −NO_2_ groups at −R^1–5^ positions in **1**_(**C**, **E**, **G**, **I** or **K**, respectively)_ increases the minimum ESP value localized on the N atoms associated with N^···.^Na^+^ interactions (Table 2). This accounts for the less attractive Δ*V*_elstat_ energy term in the **1**_(**C**, **E**, **G**, **I,** or **K**)_^···.^Na^+^ bonds compared to the **1**_**A**_^···.^Na^+^ interaction (Table 1). The additional ESP minimum value present in the oxygen atoms of the −NO_2_ groups in **1**_**G**_, contributing to the O^···.^Na^+^ interaction, does not relevantly enhance the recognition of Na^+^ by **1**_**G**_.

The NOCV method provides insights into significant orbital interactions, such as those between **1**_**A**_ and Na^+^. This is achieved by decomposing the interaction into pairwise contributions from the most relevant molecular orbitals. Each pairwise orbital interaction of a specific chemical bond can be visualized through deformation density channels, Δρ_k_(r), where red regions represent electron density outflow and blue regions depict inflow. The NOCV method also quantifies the energetic contribution (Δ*E*_oi,k_) of each density deformation channel (Δρ_k_) to the overall orbital interaction energy (Δ*E*_oi_).^29,30^

**Figure 5:**
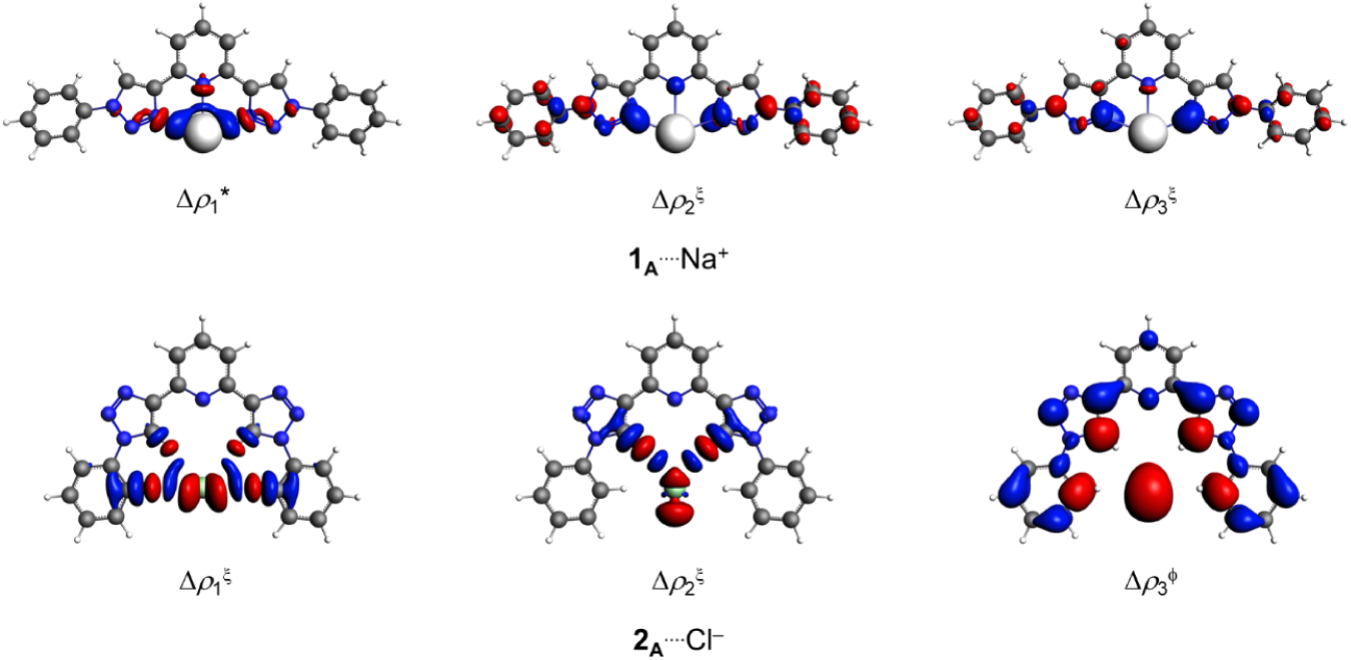
Surface plots of the first density deformation channels, Δρ_1–3_, with isovalues of ϕ = 0.0001, ξ = 0.0005, and * = 0.0010 au The red and blue regions represent electron density outflow and inflow, respectively, for the **1**_**A**_^···.^Na^+^ and **2**_**A**_^···.^Cl^–^ complexes.

The main density deformation channels for the **1**_**A**–**K**_^···.^(Li^+^, Na^+^, or K^+^) complexes are presented in Figures 5, S2, S4 and S5, with their corresponding Δ*E*_oi,1–3_ values listed in Table 1. These channels indicate that the primary orbital interactions in the **1**_**A**–**K**_^···.^(Li^+^, Na^+^, or K^+^) bonds are π and, predominantly, σ-type interactions involving (O or, chiefly, N)^···.^(Li^+^, Na^+^, or K^+^).

The Δ*E*_oi_ energy term is related to the sum of individual orbital interaction energies (Δ*E*_oi,1–3_) from the most significant density deformation channels (Δρ_1–3_). This analysis rationalizes the trend of increasingly favorable orbital interactions in the **1**_**A**_^···.^cation complexes as the cation size decreases from K^+^ → Na^+^ → Li^+^. The energy contributions from the main density deformation channels in the **1**_**B**–**K**_^···.^Na^+^ complexes generally show similar or more favorable values compared to the **1**_**A**_^···.^Na^+^ structure, as reflected by the Δ*E*_oi_ energy in Table 1.

In addition, the topological analysis of electron density, performed via the QTAIM method, revealed bond critical points (BCPs)^31^ between the **1**_**A**–**K**_ receptors and the cations (Li^+^, Na^+^, or K^+^), as shown in Figures 4b, S8, and Table S1. The ratio of kinetic energy density (*G*_b_) to potential energy density (*V*_b_), denoted as −*G*_b_/*V*_b_, at the BCPs for the (O or, primarily, N)^···.^(Li^+^, Na^+^, or K^+^) interactions is greater than 1.0, indicating the predominantly noncovalent nature of these bonds.^32^

The largest electron density (ρ_b_) value at the N^···.^Li^+^ BCP in the **1**_**A**_^···.^Li^+^ complex is larger than that at the N^···.^Na^+^ BCP of **1**_**A**_^···.^Na^+^ and the N^···.^K^+^ BCP of **1**_**A**_^···.^K^+^, in agreement with the more attractive Δ*E*_oi_ energy observed in the **1**_**A**_^···.^Li^+^ interaction compared to **1**_**A**_^···.^Na^+^ and **1**_**A**_^···.^K^+^ (Table 1). Furthermore, the total electron density at the (O or, chiefly, N)^···.^Na^+^ BCPs in the **1**_**B**–**K**_^···.^Na^+^ complexes is generally similar to or more attractive than that of the **1**_**A**_^···.^Na^+^ complex (Table S1). These findings align with the trends observed in the Δ*E*_oi_ energies for the **1**_**B**–**K**_^···.^Na^+^ complexes relative to **1**_**A**_^···.^Na^+^ (Table 1).

### Anionic Recognition

The EDA results related to the recognition of F^–^, Cl^–^, or Br^–^ anions by the **2**_**A**–**K**_ receptors are presented in Table 3. The analysis reveals comparable contributions from the Δ*V*_elstat_ (42–56%) and Δ*E*_oi_ (38–58%) components. In contrast, the Δ*E*_disp_ term has a smaller contribution, accounting for only 0–10% of the total interaction energy (Δ*V*_elstat_ + Δ*E*_oi_ + Δ*E*_disp_). These results indicate that the interactions between the **2**_**A**–**K**_ receptors and the anions exhibit a predominantly noncovalent or polar covalent character.

**Table 3:** Analysis of the Bonding Situations between the Receptors (**2**_**A**–**K**_) and Anions (F^–^, Cl^–^, or Br^–^) Using the EDA-NOCV Methodology^a^,^b^,^c^

Complex	Δ*E*_int_	Δ*V*_elstat_	Δ*E*_Pauli_	Δ*E*_oi_	Δ*E*_disp_	Δ*E*_oi,1_	Δ*E*_oi,2_	Δ*E*_oi,3_
**2**_**A**_^···.^F^–^	–88.29	–97.72 (42)	145.63	–135.23 (58)	–0.96 (0)	–93.28	–7.70	–14.14
**2**_**A**_^···.^Cl^–^	–40.19	–33.92 (50)	27.85	–28.65 (42)	–5.46 (8)	–9.18	–4.98	–2.30
**2**_**A**_^···.^Br^–^	–35.29	–35.97 (50)	36.40	–28.82 (40)	–6.90 (10)	–9.52	–4.84	–2.08
**2**_**B**_^···.^Cl^–^	–37.64	–32.30 (48)	29.29	–29.02 (43)	–5.62 (8)	–9.11	–4.87	–2.32
**2**_**C**_^···.^Cl^–^	–52.90	–48.82 (56)	33.99	–32.20 (37)	–5.86 (7)	–10.24	–2.67	–5.74
**2**_**D**_^···.^Cl^–^	–49.07	–43.68 (55)	30.12	–29.75 (38)	–5.76 (7)	–9.85	–4.81	–1.78
**2**_**E**_^···.^Cl^–^	–55.91	–49.65 (54)	36.63	–36.00 (39)	–6.90 (7)	–10.75	–6.85	–3.75
**2**_**F**_^···.^Cl^–^	–48.50	–46.47 (54)	37.95	–33.49 (39)	–6.49 (8)	–11.76	–4.36	–3.84
**2**_**G**_^···.^Cl^–^	–46.30	–38.32 (51)	28.36	–28.64 (38)	–7.70 (10)	–6.59	–6.59	–1.97
**2**_**H**_^···.^Cl^–^	–37.69	–33.36 (48)	31.28	–29.92 (43)	–5.69 (8)	–9.54	–5.04	–2.39
**2**_**I**_^···.^Cl^–^	–50.31	–47.25 (55)	34.95	–32.09 (38)	–5.91 (7)	–9.92	–5.81	–2.72
**2**_**J**_^···.^Cl^–^	–38.32	–33.24 (49)	29.82	–29.28 (43)	–5.63 (8)	–9.23	–4.91	–2.57
**2**_**K**_^···.^Cl^–^	–45.84	–41.26 (53)	31.44	–30.31 (39)	–5.70 (7)	–9.45	–2.54	–5.49

a The energy unit is kcal mol^–1^.

b Δ*E*_int_ = Δ*V*_elstat_ + Δ*E*_Pauli_ + Δ*E*_oi_ + Δ*E*_disp_.

c Values in parentheses represent the percentage of each stabilizing contribution (Δ*V*_elstat_ + Δ*E*_oi_ + Δ*E*_disp_ = 100%).

The **2**_**A**_ molecule exhibits a more attractive interaction with the F^–^ anion compared to Cl^–^ and Br^–^, as evidenced by its more favorable interaction energy. This is primarily due to more stabilizing Δ*V*_elstat_ and Δ*E*_oi_ energies, despite the presence of more repulsive Δ*E*_Pauli_ and less attractive Δ*E*_disp_ energy in the **2**_**A**_^···.^F^–^ complex compared to the **2**_**A**_^···.^Cl^–^ and **2**_**A**_^···.^Br^–^ compounds.

The **2**_**A**–**K**_ compounds were studied to assess the impact of electron-donating (−NH_2_) or electron-accepting (−NO_2_) groups at different positions on Cl^–^ anion recognition (Figure 2). Substituting the hydrogen atoms at the −R,^1^ −R,^4^ and −R^5^ positions of **2**_**A**_ with −NH_2_ groups yielding the **2**_(**B**, **H,** or **J**, respectively)_ reduces Cl^–^ recognition (Figure 3). The less attractive **2**_(**B**, **H,** or **J**)_^···.^Cl^–^ interactions, compared to the **2**_**A**_^···.^Cl^–^ bond, are primarily due to less favorable Δ*V*_elstat_ energy or because of the more repulsive Δ*E*_Pauli_ energetic term. The Δ*E*_oi_ and Δ*E*_disp_ contributions remain relatively similar across the **2**_(**A**, **B**, **H,** or **J**)_^···.^Cl^–^ complexes. As an exception, the **2**_**H**_^···.^Cl^–^ bond has a more favorable Δ*E*_oi_ energy component than the **2**_**A**_^···.^Cl^–^ interaction.

The substitution of −H atoms in the **2**_**A**_ receptor with −NH_2_ groups at the −R^2^ and −R^3^ positions, forming the **2**_(**D** or **F**, respectively)_ molecules, enhances the interaction with the Cl^–^ anion (Figures 2 and 3). The more stabilizing interaction with Cl^–^ arises from more favorable Δ*V*_elstat_ and Δ*E*_oi_ energy terms, despite an increase in Δ*E*_Pauli_. Δ*E*_disp_ remains comparable between **2**_(**A** or **D**)_^···.^Cl^–^ complexes but is more attractive in the **2**_**F**_^···.^Cl^–^ structure compared to **2**_**A**_^···.^Cl^–^ molecules.

The substitution of −H atoms in **2**_**A**_ with −NO_2_ groups at the −R^1–5^ positions, forming the **2**_(**C**, **E**, **G**, **I,** or **K**, respectively)_ compounds, enhances Cl^–^ binding (Figures 2 and 3). This is largely due to similar or more attractive Δ*V*_elstat_, Δ*E*_oi_ and Δ*E*_disp_ terms, although there is comparable or greater Pauli repulsion in the **2**_(**C**, **E**, **G**, **I,** or **K**)_^···.^Cl^–^ interactions compared to **2**_**A**_^···.^Cl^–^.

The ESP surfaces of the isolated **2**_**A**–**K**_ molecules reveal regions of low electron density around (i) hydrogen atoms of −NH_2_ and, principally, −CH groups; and (ii) carbon atoms attached to −NO_2_ groups (Figures 4a and S1). High electron density is observed around the isolated F^–^, Cl^–^, and Br^–^ anions (Figure 4a). The decreasing trend in minimum ESP values (Br^–^ → Cl^–^ → F^–^, Table 2) explains the more favorable Δ*V*_elstat_ energy in the **2**_**A**_^···.^F^–^ interaction compared to the **2**_**A**_^···.^Cl^–^ and **2**_**A**_^···.^Br^–^ bonds.

The replacement of hydrogen atoms with −NH_2_ groups at the −R^1–5^ positions in **2**_**A**_ (forming **2**_(**B**, **D**, **F**, **H,** or **J**, respectively)_) decreases the maximum ESP values related to H atoms in C–H^···.^Cl^–^ interactions (Figure 2 and Table 2). This reduction helps explain the close or less favorable Δ*V*_elstat_ energy in **2**_(**B**, **H,** or **J**)_^···.^Cl^–^ complexes compared to **2**_**A**_^···.^Cl^–^ (Figure 3). Specifically, the more favorable Δ*V*_elstat_ in the **2**_(**D** or **F**)_^···.^Cl^–^ complexes, compared to **2**_**A**_, is due to additional N–H^···.^Cl^–^ interactions introduced by the −NH_2_ groups at these positions.

The substitution of −H with −NO_2_ groups at the −R^1,2,4,5^ positions in the **2**_**A**_ receptor, forming the **2**_(**C**, **E**, **I,** or **K**, respectively)_ molecules, leads to an increase in the maximum ESP values associated with hydrogen atoms involved in C–H^···.^Cl^–^ interactions (Figure 2 and Table 2). As a special case, there is a decrease in in the maximum ESP values related to hydrogen atoms involved in C–H^···.^Cl^–^ bonds present in the **2**_**G**_ compound compared to the **2**_**A**_ molecule. However, the **2**_**G**_ receptor exhibits a maximum ESP region around the carbon atom bonded to the −NO_2_ group (Table 2), which contributes to a favorable O_2_NC^···.^Cl^–^ interaction in the **2**_**G**_^···.^Cl^–^ complex. This information helps explain the more attractive Δ*V*_elstat_ energy in the **2**_(**C**, **E**, **G**, **I,** or **K**)_^···.^Cl^–^ bonds compared to the **2**_**A**_^···.^Cl^–^ interaction (Figure 3).

The main density deformation channel surface plots for the **2**_**A**–**K**_^···.^(F^–^, Cl^–^, or Br^–^) molecules are displayed in Figures 5, S6 and S7. The corresponding Δ*E*_oi,1–3_ values are provided in Table 3. The deformation channels (Δρ_1–3_) suggest that the dominant orbital interactions in the **2**_**A**–**K**_^···.^(F^–^, Cl^–^, or Br^–^) complexes involve electron donation from the anions’ lone pairs (F^–^, Cl^–^, or Br^–^)(n) to the π* or predominantly σ* orbitals of (C–H, HN–H, or −CNO_2_).

The Δ*E*_oi_ energy, representing the sum of individual orbital interaction energies Δ*E*_oi,1–3_ (Table 3), indicates more favorable orbital interactions in the **2**_**A**_^···.^anion bonds as the anion size decreases from Br^–^ and Cl^–^ to F^–^. The trends observed for Δ*E*_oi,1–3_ generally align with those seen for the overall Δ*E*_oi_ energies in the **2**_(**B**–**K**)_^···.^Cl^–^ complexes relative to **2**_**A**_^···.^Cl^–^. Specifically, the Cl^–^(n) → (C–H or HN–H)(π* or mostly σ*) orbital interactions are similar or more favorable for **2**_(**B**–**F** or **H**–**K**)_^···.^Cl^–^ compared to **2**_**A**_^···.^Cl^–^. On the other hand, the Cl^–^(n) → (C–H or −CNO_2_)(π* or mostly σ*) orbital interactions in **2**_**G**_^···.^Cl^–^ have a less attractive energy compared to Cl^–^(n) → (C–H)(π* or mostly σ*) orbital interactions in **2**_**A**_^···.^Cl^–^.

The QTAIM method reveals BCPs between the **2**_**A**–**K**_ compounds and the anions (F^–^, Cl^–^, or Br^–^) (Figure S9 and Table S2). Generally, the −G_b_/V_b_ ratios at BCPs associated with (N or primarily C)–H^···.^(F^–^, Cl^–^, or Br^–^) and O_2_NC^···.^Cl^–^ bonds exceed 1.0, indicating that these interactions are predominantly noncovalent.^32^ In some cases, such as a C–H^···.^F^–^ interaction in the **2**_**A**_^···.^F^–^ complex, the −G_b_/V_b_ value falls between 0.5 and 1.0, suggesting a partially covalent nature.^32^

The sum of electron density at the BCPs (Δρ_b_) for C–H^···.^F^–^ bonds in **2**_**A**_^···.^F^–^ is larger than that for C–H^···.^Cl^–^ (**2**_**A**_^···.^Cl^–^) and C–H^···.^Br^–^ BCPs (**2**_**A**_^···.^Br^–^) (Table S2). These observations align with the more favorable Δ*E*_oi_ energy component in the **2**_**A**_^···.^F^–^ complex compared to the **2**_**A**_^···.^Cl^–^ and **2**_**A**_^···.^Br^–^ complexes (Table 3).

Overall, changes in Δρ_b_ values associated with (N or predominantly C)–H^···.^Cl^–^ and O_2_NC^···.^Cl^–^BCPs are consistent with trends in Δ*E*_oi_ energy for the **2**_**A**–**K**_^···.^Cl^–^ chemical bonding scenarios (Tables S2 and 3). The substitution of −H with −NH_2_ groups at the −R^1–5^ positions in **2**_**A**_, resulting in the **2**_(**B**, **D**, **F**, **H**, or **J**, respectively)_ compounds, does not significantly alter or increase the Δρ_b_ values at (N or predominantly C)–H^···.^Cl^–^ BCPs (in agreement with the close or more attractive Δ*E*_oi_ energy) in the **2**_(**B**, **D**, **F**, **H,** or **J**)_^···.^Cl^–^ complexes concerning to the **2**_**A**_^···.^Cl^–^ structure.

The substitution of hydrogen atoms at the −R^1,2,4,5^ positions of **2**_**A**_ with −NO_2_ groups, forming **2**_(**C**, **E**, **I,** or **K**, respectively)_ molecules, improves the Δρ_b_ associated with C–H^···.^Cl^–^ BCPs. This enhancement correlates with the more favorable Δ*E*_oi_ energetic term in the **2**_(**C**, **E**, **I,** or **K**)_^···.^Cl^–^ bonds compared to the **2**_**A**_^···.^Cl^–^ interaction (Tables S2 and 3). However, the presence of −NO_2_ groups at the −R^3^ positions in the **2**_**G**_ structure decreases the Δρ_b_ value at the C–H^···.^Cl^–^ and O_2_NC^···.^Cl^–^ BCPs compared to C–H^···.^Cl^–^ BCPs at **2**_**A**_^···.^Cl^–^ molecules. This aligns with the less attractive Δ*E*_oi_ energy in the **2**_**G**_^···.^Cl^–^ bond relative to the **2**_**A**_^···.^Cl^–^ interaction.

### Cationic X Anionic Recognition

To clarify whether the **1**_**A**–**K**_ conformers preferentially recognize cations compared to the **2**_**A**–**K**_ conformers’ interactions with anions, the interactions between **1**_**A**–**K**_^···.^(Li^+^, Na^+^, or K^+^) and **2**_**A**–**K**_^···.^(F^–^, Cl^–^, or Br^–^) were analyzed. The study focused on comparing conformers with similar structural modifications and ions from the same period of the periodic table, for instance, **1**_**A**_^···.^Li^+^ versus **2**_**A**_^···.^F^–^.

The EDA results demonstrate that the cationic recognition by the **1**_**A**–**K**_ molecules is more favorable than anionic recognition by the **2**_**A**–**K**_ compounds. In general, this is attributed to the more attractive Δ*V*_elstat_ energy and/or a less repulsive Δ*E*_Pauli_ component in the **1**_**A**–**K**_^···.^(Li^+^, Na^+^, or K^+^) bonds compared to the **2**_**A**–**K**_^···.^(F^–^, Cl^–^, or Br^–^) interactions (Tables 1 and 3). The stronger electrostatic interactions in the **1**_**A**–**K**_^···.^cation complex can be explained by the larger absolute maximum ESP values in isolated cations compared to the absolute minimum ESP values in isolated anions (Table 2).

In addition, the NOCV methodology reveals that the sum of the energy contributions from the most relevant density deformation channels indicates that π and, predominantly, σ orbital interactions involving (O or chiefly N)^···.^(Li^+^, Na^+^, or K^+^) in the **1**_**A**–**K**_^···.^(Li^+^, Na^+^ or K^+^) complexes are less attractive than the (F^–^, Cl^–^, or Br^–^)(n) → (C–H, HN–H, or −CNO_2_)(π* or mostly σ*) orbital interactions in the **2**_**A**–**K**_^···.^(F^–^, Cl^–^, or Br^–^) complexes (Tables 1 and 3). Overall, this finding further explains the less favorable or close Δ*E*_oi_ energy term for the **1**_**A**–**K**_^···.^(Li^+^, Na^+^, or K^+^) interactions compared to the **2**_**A**–**K**_^···.^(F^–^, Cl^–^, or Br^–^) bonds.

Moreover, QTAIM analysis shows that the BCPs for the (O or primarily N)^···.^(Li^+^, Na^+^, or K^+^) interactions in the **1**_**A**–**F** or **H**–**K**_^···.^(Li^+^, Na^+^, or K^+^) complexes exhibit lower Δρ_b_ values than the (N or chiefly C)–H^···.^(F^–^, Cl^–^, or Br^–^) BCPs in the **2**_**A**–**F** or **H**–**K**_^···.^(F^–^, Cl^–^, or Br^–^) complexes (Tables S1 and S2). These lower Δρ_b_ values align with the orbital interaction trends observed. Exceptionally, there is a larger Δρ_b_ value (in agreement with the more attractive Δ*E*_oi_ energy) associated with C–H^···.^Cl^–^ and O_2_NC^···.^Cl^–^ BCPs of the **2**_**G**_^···.^Cl^–^ structure compared to the (O or primarily N)^···.^Na^+^ BCPs of the **1**_**G**_^···.^Na^+^ complex

## Conclusions

The findings of this investigation reveal that the **1**_**A**_ conformer preferentially interacts with cations, with the interaction becoming more favorable as the cation size decreases (K^+^ → Na^+^ → Li^+^). This trend arises from more attractive electrostatic and orbital (π and mainly σ) N^···.^cation interactions in the **1**_**A**_^···.^Li^+^ complex compared to the **1**_**A**_^···.^Na^+^ and **1**_**A**_^···.^K^+^ structures, respectively.

Substituting hydrogen atoms with electron-donating groups (−NH_2_) in the **1**_**A**_ receptor enhances the recognition of Na^+^ ions. This improvement is primarily due to more favorable electrostatic N^···.^Na^+^ bonds in the **1**_(**B**, **D**, **F**, **H,** or **J**)_^···.^Na^+^ complexes compared to the **1**_**A**_^···.^Na^+^ interaction. In contrast, substituting hydrogen atoms in **1**_**A**_ with electron-accepting groups (−NO_2_) leads to less stabilizing N^···.^Na^+^ interactions in the **1**_(**C**, **E**, **I,** or **K**)_^···.^Na^+^ complexes relative to **1**_**A**_.

For anion recognition, the **2**_**A**_ conformer interacts more favorably with smaller anions (Br^–^ → Cl^–^ → F^–^) due to stronger electrostatic and anion(n) → C–H(π* or largely σ*) orbital interactions in the **2**_**A**_^···.^F^–^ structure compared to **2**_**A**_^···.^Cl^–^ and **2**_**A**_^···.^Br^–^.

Selective substitution of hydrogen atoms by −NH_2_ groups at the −R^2.3^ positions of **2**_**A**_ (yielding **2**_**D**_ and **2**_**F**_) enhances Cl^–^ recognition. These specific substitutions create additional electrostatic and anion(n) → N–H(π* or largely σ*) orbital interactions. In contrast, other −H → −NH_2_ substitutions at the −R^1,4,5^ positions (yielding **2**_**B**_, **2**_**H**_, and **2**_**J**_) reduce Cl^–^ recognition, mainly due to greater Pauli repulsive energy in the **2**_(**B**, **H,** or **J**)_^···.^Cl^–^ structures compared to **2**_**A**_^···.^Cl^–^ molecules.

Substituting −H atoms with −NO_2_ groups at the −R^1–5^ positions of **2**_**A**_ consistently improves anion recognition. This enhancement can be explained by the similar or more attractive electrostatic interactions, Cl^–^(n) → (C–H, HN–H, or −CNO_2_)(π* or mostly σ*) orbital interactions, and dispersion interactions in the **2**_(**C**, **E**, **G**, **I,** or **K**)_^···.^Cl^–^ molecules compared to the **2**_**A**_^···.^Cl^–^ complex.

Finally, the **1**_**A**–**K**_ conformers exhibit a stronger ability to recognize cations (Li^+^, Na^+^, or K^+^) compared to the **2**_**A**–**K**_ conformers’ interactions with anions (F^–^, Cl^–^, or Br^–^). This difference is attributed to more attractive electrostatic and/or less Pauli repulsive energies in the **1**_**A**–**K**_^···.^(Li^+^, Na^+^, or K^+^) structures relative to **2**_**A**–**K**_^···.^(F^–^, Cl^–^, or Br^–^) complexes.

These insights could be leveraged to design materials capable of recognizing either cations or anions using the same base structure. By making specific structural modifications, ionic recognition can be enhanced. The presence of electron-donating groups in the receptor structure favors cation recognition, while electron-accepting groups promote more attractive anion recognition. Importantly, cations tend to be preferentially recognized by the base molecules in comparison to anions.

## Computational Methods

The geometry optimization of all molecules was performed without any geometric constraints, and vibrational frequency calculations were carried out using the BLYP^33−35^ functional, including dispersion corrections (Grimme’s D3(BJ) method with Becke–Johnson damping).^26^ The basis set employed for these calculations was Def2-TZVP.^36^ The RIJCOSX^37,38^ approximation was applied to accelerate the calculations, and the Coulomb integrals were approximated using the RI–J^37^ method with the Def2/J^39^ auxiliary basis set. To ensure that all optimized geometries correspond to minimum energy states, vibrational frequency analysis was conducted to confirm the absence of imaginary frequencies, which is essential for verifying the validity of the computational model chosen. All calculations were performed using the ORCA software package.^40^

The choice of the BLYP-D3(BJ)/Def2-TZVP level of theory for geometry optimization is supported by literature recommendations for systems with significant noncovalent interactions.^27^ The ESP surfaces and wave functions used in the QTAIM analysis were calculated using Gaussian 16 (Revision A.03) at the BLYP-D3(BJ)/Def2-TZVP level of theory.^41^ ESP surface analysis was conducted using Multiwfn 3.7 with an electron density isovalue of 0.001 au.^42,43^ The topological analysis of the electron density was performed via the QTAIM^31,44^ approach using AIMAll (Version 17.01.25).^45^

The bonding mechanism of the noncovalent interactions was investigated using the EDA^25^-NOCV^29,30^ methodology. These calculations were carried out using the Amsterdam Density Functional (ADF)^46,47^ software at the BLYP-D3(BJ) level of theory with the TZ2P^48^ basis set. Scalar relativistic corrections were incorporated using the zero-order regular approximation (ZORA).^49^ The ZORA-BLYP-D3(BJ)/TZ2P computational model was found to be suitable for shedding light on the bonding mechanisms governing noncovalent interactions.^50,51^

## Supporting Information Available

MEP, NOCV, and QTAIM results for selected structures and optimized Cartesian coordinates of all complexes investigated (PDF)
