# Supplementary material for: Ionic Recognition Controlled by Conformational Change: A DFT Investigation
Source: ACS Omega. 2025 Apr 16;10(16):16114–22. doi: 10.1021/acsomega.4c09597 (PMC12044477; doi:10.1021/acsomega.4c09597)
Supplement: Supplementary file 1 — ao4c09597_si_001.pdf [file ao4c09597_si_001.pdf]

Ionic Recognition Controlled by Conformational Change: A DFT Investigation

Renato Pereira Orenha,*[a] Ana Lívia de Oliveira Andrade,[a] Renato Gonçalves Rocha,[a] Alvaro Muñoz–Castro,[b] Thiago Ferreira Santos,[c] Maurício Jeomar Piotrowski,[c] Giovanni Finoto Caramori,*[d] and Renato Luis Tame Parreira*[a]

[a] Núcleo de Pesquisas em Ciências Exatas e Tecnológicas, Universidade de Franca, Av. Dr. Armando Salles Oliveira 201, Franca, SP, 14404–600, Brazil. Email: rpo9@hotmail.com / renato.parreira@unifran.edu.br

[b] Facultad de Ingeniería, Arquitectura y Diseño, Universidad San Sebastián, Bellavista 7, Santiago, 8420524, Chile.

[c] Department of Physics, Federal University of Pelotas, Pelotas, RS, 96010–900, Brazil.

[d] Departamento de Química, Universidade Federal de Santa Catarina, Campus Universitário Trindade, CP 476, Florianópolis, SC, 88040–900, Brazil. Email: giovanni.caramori@ufsc.br

Supplementary Material

Summary

	Page
**Figure S1.** Electrostatic potential surfaces mapped onto an electronic density of 0.050 a.u. [ranging from 0.100 a.u. (red) to 0.250 a.u. (blue)] for the $\mathbf{1}_{\mathbf{B–K}}$ / $\mathbf{2}_{\mathbf{B–K}}$ receptors.	S3
**Figure S2.** Surface plots of the first density deformation channels, $\Delta\rho_{1–3}$, with isovalues of ϕ = 0.0001, ξ = 0.0005 and * = 0.0010 a.u. The red and blue regions represent electron density outflow and inflow, respectively, for the $\mathbf{1}_{\mathbf{A}}\cdots(Li^+$, or $K^+)$ complexes.	S4
**Figure S3.** Surface plots of the first density deformation channels, $\Delta\rho_{1–3}$, with isovalues of ϕ = 0.0001, ξ = 0.0005 and * = 0.0010 a.u. The red and blue regions represent electron density outflow and inflow, respectively, for the $\mathbf{2}_{\mathbf{A}}\cdots(F^-$, or $Br^-)$ complexes.	S5
**Figure S4.** Surface plots of the first density deformation channels, $\Delta\rho_{1–3}$, with isovalues of ξ = 0.0005 and * = 0.001 a.u. The red and blue regions represent electron density outflow and inflow, respectively, for the $\mathbf{1}_{\mathbf{B–G}}\cdots Na^+$ complexes.	S6
**Figure S5.** Surface plots of the first density deformation channels, $\Delta\rho_{1–3}$, with isovalues of ξ = 0.0005 and * = 0.001 a.u. The red and blue regions represent electron density outflow and inflow, respectively, for the $\mathbf{1}_{\mathbf{I–K}}\cdots Na^+$ complexes.	S7
**Figure S6.** Surface plots of the first density deformation channels, $\Delta\rho_{1–3}$, with isovalues of ϕ = 0.0001 and ξ = 0.0005 a.u. The red and blue regions represent electron density outflow and inflow, respectively, for the $\mathbf{2}_{\mathbf{B–F}}\cdots Cl^-$ complexes.	S8
**Figure S7.** Surface plots of the first density deformation channels, $\Delta\rho_{1–3}$, with isovalues of ϕ = 0.0001 and ξ = 0.0005 a.u. The red and blue regions represent electron density outflow and inflow, respectively, for the $\mathbf{2}_{\mathbf{G–K}}\cdots Cl^-$ complexes.	S9

**Figure S8.** Topological maps showing bond paths (continuous or dashed lines connecting the cores) and bond critical points (small light green points), for the $\mathbf{1_A}$···(Li^+ or, K^+) and $\mathbf{1_{B–K}}$···Na^+ complexes. Color code for atoms: H = white; Li = light purple; C = gray; N = blue; O = red; Na = purple; and K = dark purple.	S10
**Figure S9.** Topological maps showing bond paths (continuous or dashed lines connecting the cores) and bond critical points (small light green points), for the $\mathbf{2_A}$···($F^–$ or, $Br^–$) and $\mathbf{2_{B–K}}$···$Cl^–$ complexes. Color code for atoms: H = white; C = gray; N = blue; O = red; F = lemon green; Cl = green; and Br = brown.	S11
**Table S1.** Ratio of kinetic energy density (G_b) to potential energy density (V_b), expressed as $–G_b/V_b$, along with the electron density (ρ_b) at BCPs related to the interactions in the $\mathbf{1_{A–K}}$···(Li^+, Na^+, or K^+) complexes. All values are presented in atomic units (a.u.).	S12
**Table S2.** Ratio of kinetic energy density (G_b) to potential energy density (V_b), expressed as $–G_b/V_b$, along with the electron density (ρ_b) at BCPs related to the interactions in the $\mathbf{2_{A–K}}$···($F^–$, $Cl^–$, or $Br^–$) complexes. All values are presented in atomic units (a.u.).	S13
**Table S3.** Optimized Cartesian coordinates for the compounds investigated in this study, calculated using the BLYP–D3(BJ)/Def2–TZVP computational model.	S14

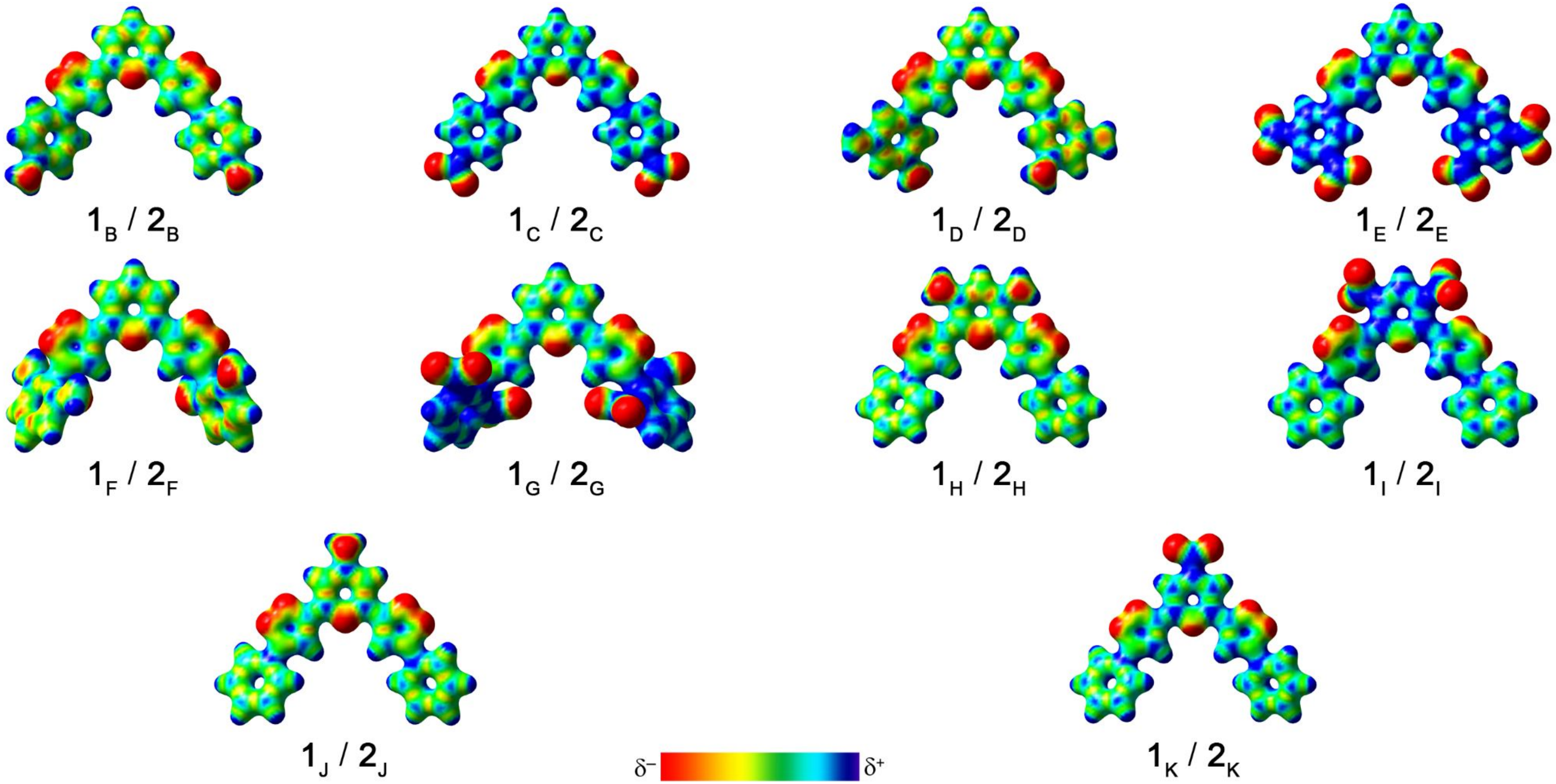

Figure S1. Electrostatic potential surfaces mapped onto an electronic density of 0.050 a.u. [ranging from 0.100 a.u. (red) to 0.250 a.u. (blue)] for the $\mathbf{1}_{\mathbf{B–K}}$ / $\mathbf{2}_{\mathbf{B–K}}$ receptors.

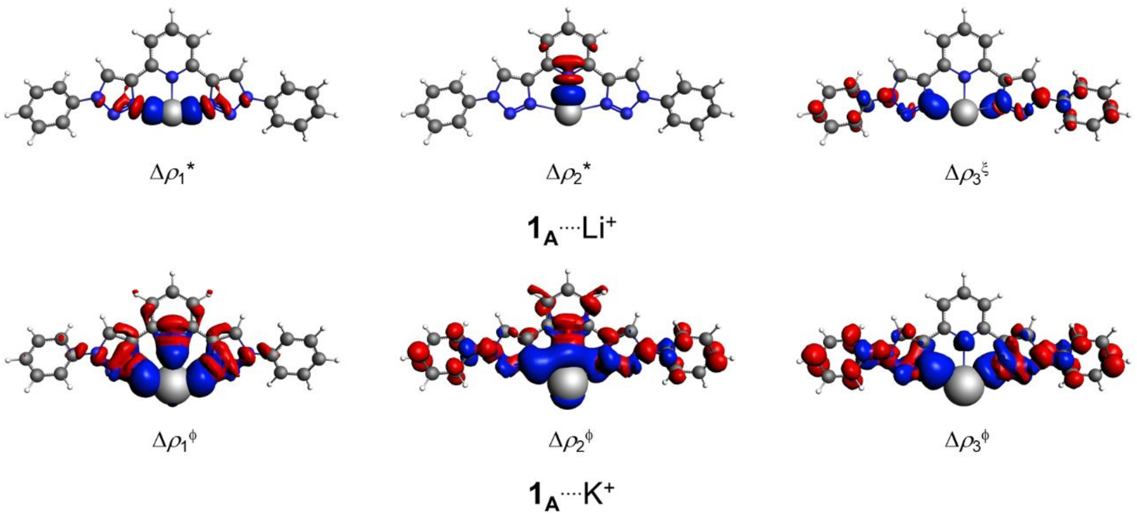

Figure S2. Surface plots of the first density deformation channels, $\Delta\rho_{1-3}$, with isovalues of ϕ = 0.0001, ξ = 0.0005 and * = 0.0010 a.u. The red and blue regions represent electron density outflow and inflow, respectively, for the $\mathbf{1_A}\cdots(Li^+$ or, $K^+)$ complexes.

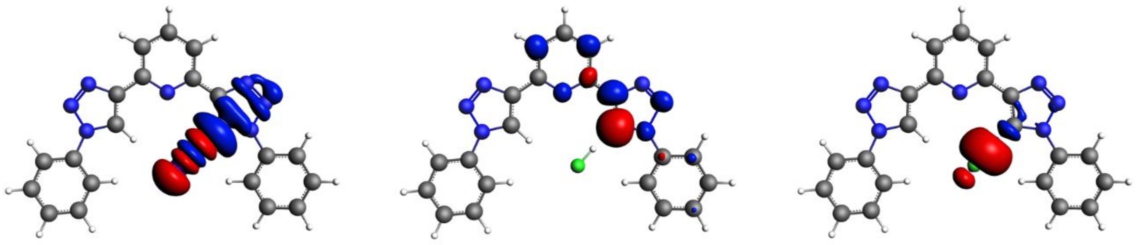

$\Delta\rho_1{}^*$ $\Delta\rho_2{}^*$ $\Delta\rho_3{}^*$

$\mathbf{2_A}\cdots F^-$

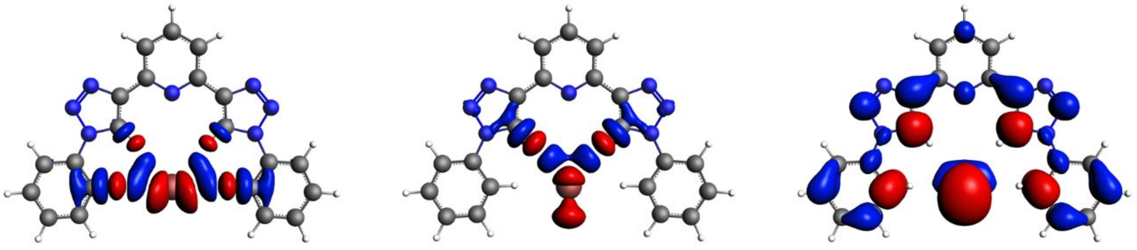

$\Delta\rho_1{}^\xi$ $\Delta\rho_2{}^\xi$ $\Delta\rho_3{}^\phi$

$\mathbf{2_A}\cdots Br^-$

Figure S3. Surface plots of the first density deformation channels, $\Delta\rho_{1-3}$, with isovalues of ϕ = 0.0001, ξ = 0.0005 and * = 0.0010 a.u. The red and blue regions represent electron density outflow and inflow, respectively, for the $\mathbf{2_A}\cdots(F^-$ or, $Br^-)$ complexes.

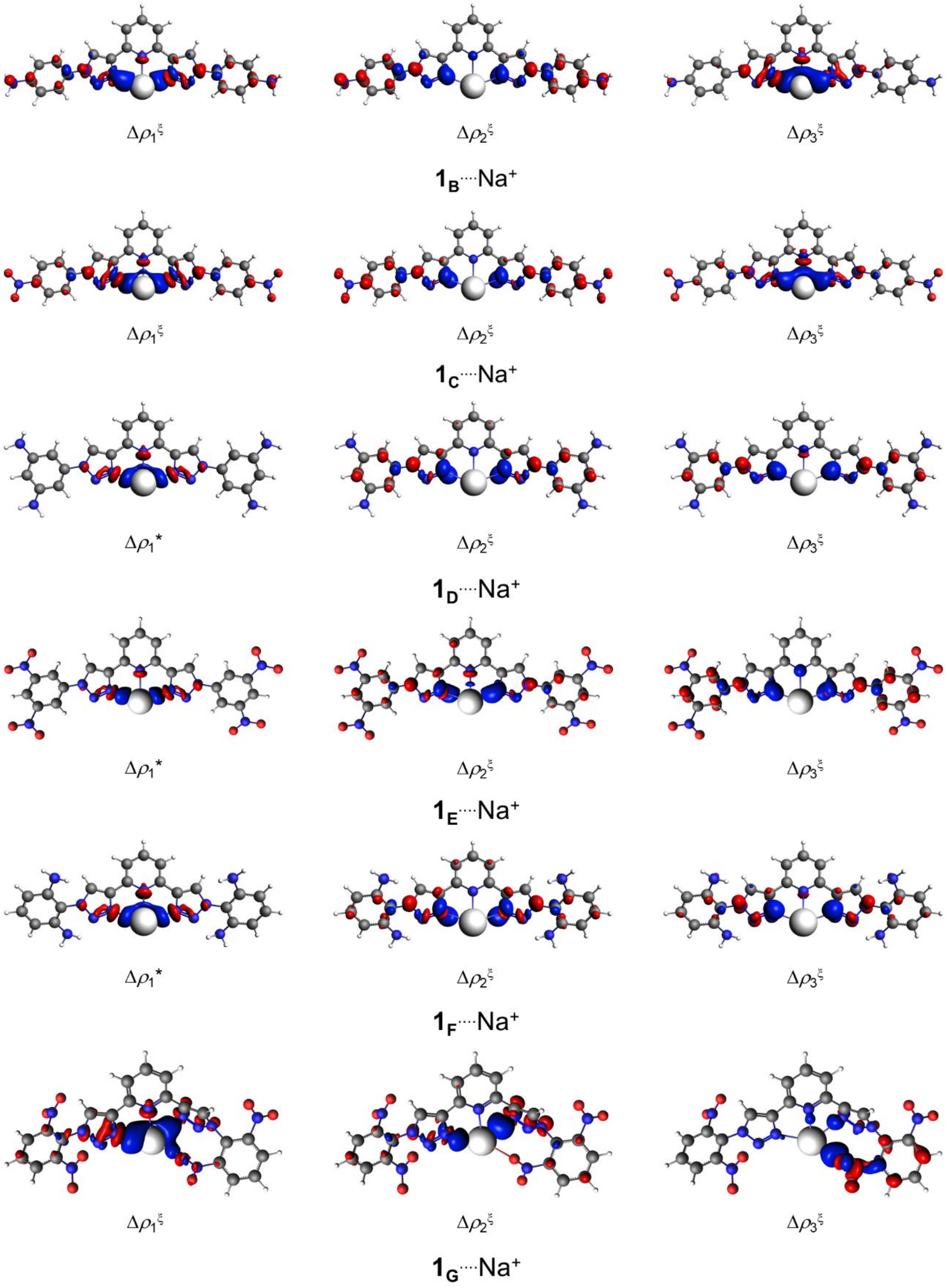

Figure S4. Surface plots of the first density deformation channels, $\Delta\rho_{1-3}$, with isovalues of ξ = 0.0005 and * = 0.001 a.u. The red and blue regions represent electron density outflow and inflow, respectively, for the $\mathbf{1}_{\mathbf{B-G}}\cdots Na^+$ complexes.

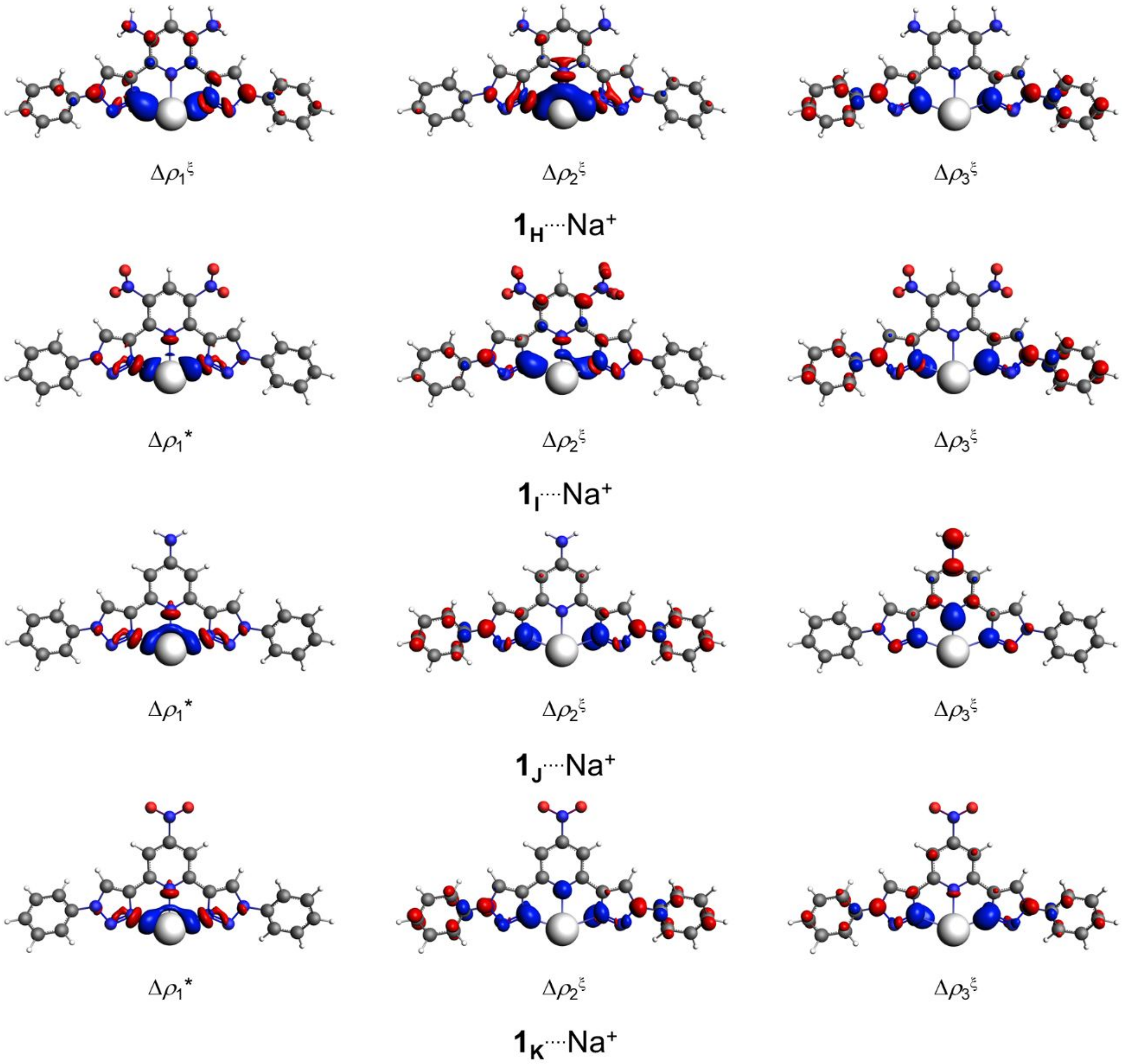

Figure S5. Surface plots of the first density deformation channels, $\Delta\rho_{1-3}$, with isovalues of ξ = 0.0005 and * = 0.001 a.u. The red and blue regions represent electron density outflow and inflow, respectively, for the $\mathbf{1_{I-K}}\cdots Na^+$ complexes.

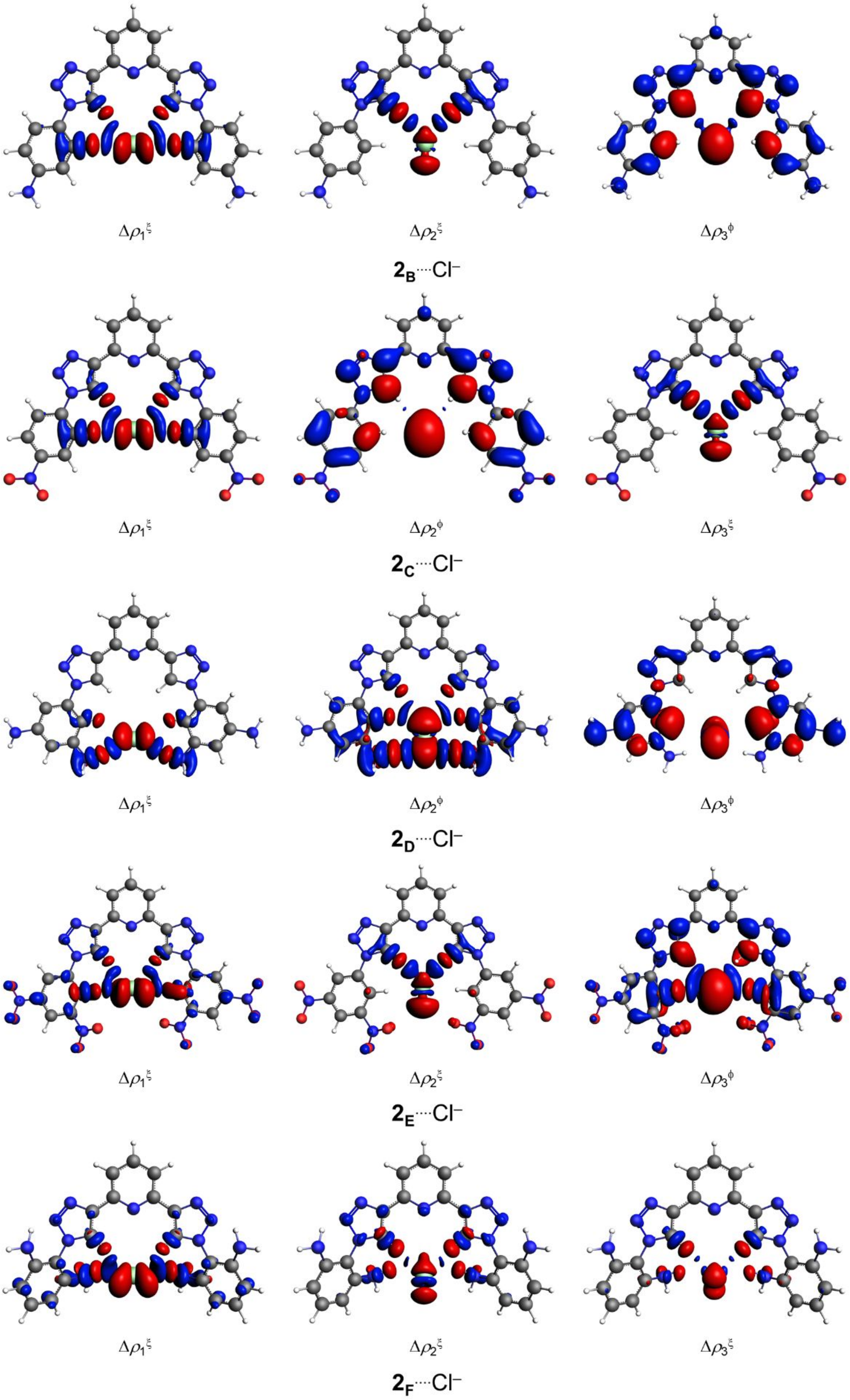

Figure S6. Surface plots of the first density deformation channels, $\Delta\rho_{1-3}$, with isovalues of ϕ = 0.0001 and ξ = 0.0005 a.u. The red and blue regions represent electron density outflow and inflow, respectively, for the $\mathbf{2_{B-F}}\cdots Cl^-$ complexes.

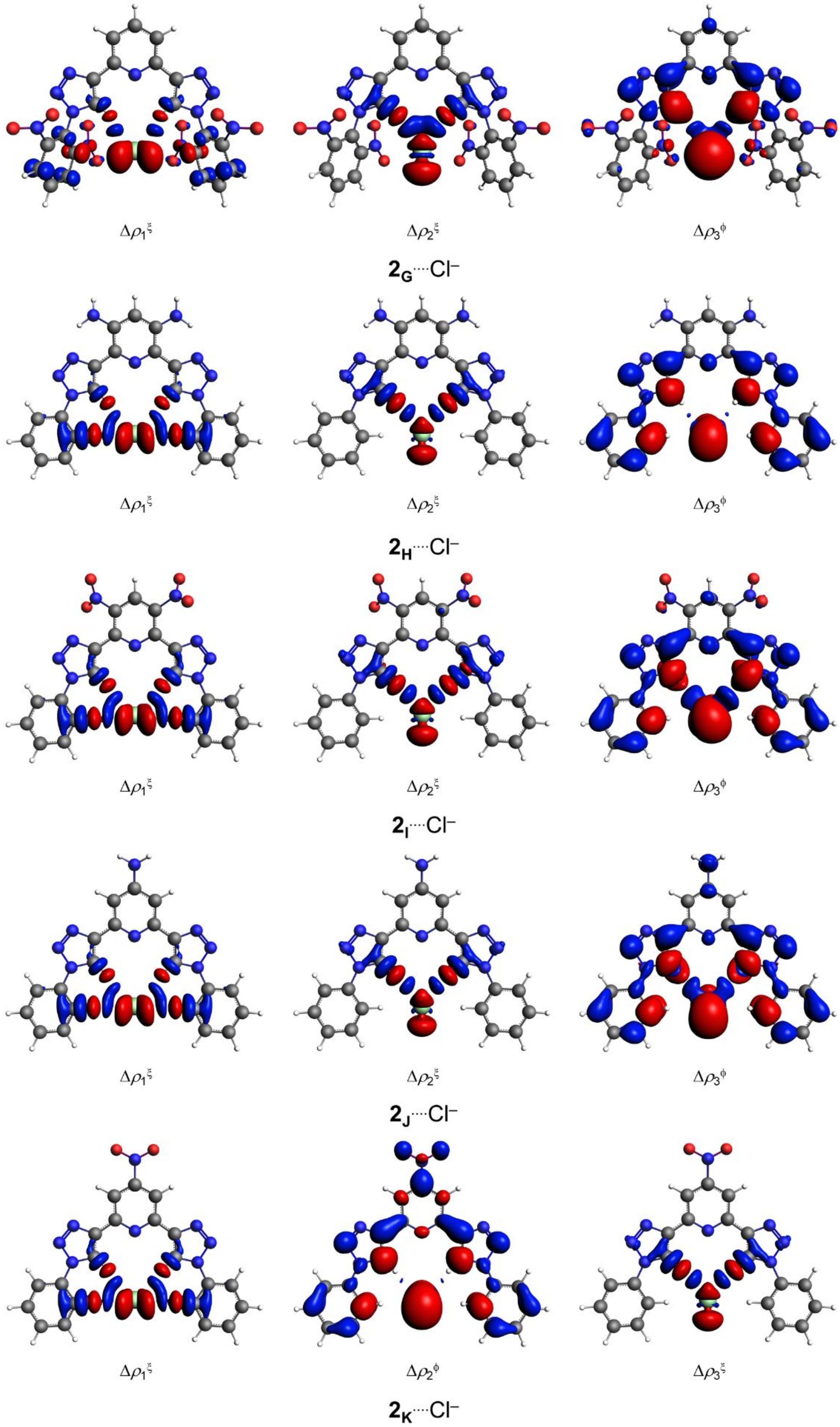

Figure S7. Surface plots of the first density deformation channels, $\Delta\rho_{1-3}$, with isovalues of ϕ = 0.0001 and ξ = 0.0005 a.u. The red and blue regions represent electron density outflow and inflow, respectively, for the $\mathbf{2_{G-K}}\cdots Cl^-$ complexes.

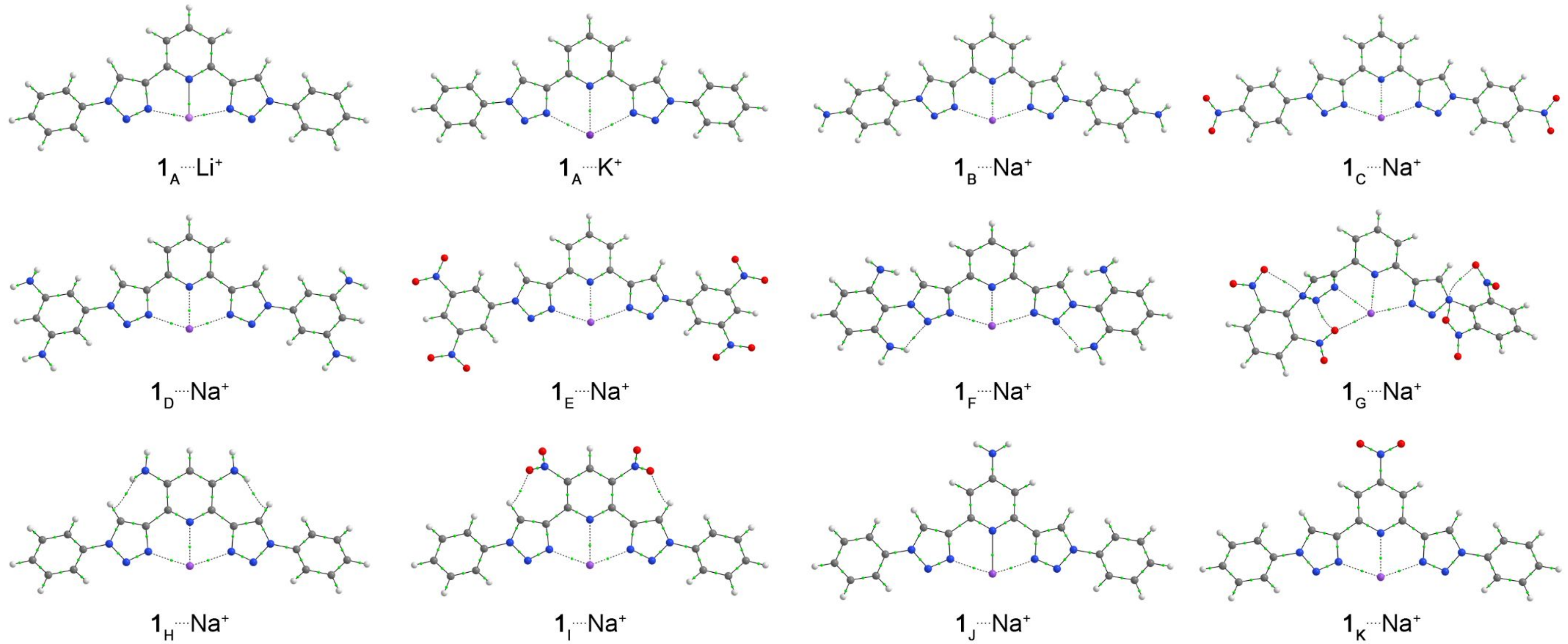

Figure S8. Topological maps showing bond paths (continuous or dashed lines connecting the cores) and bond critical points (small light green points), for the $\mathbf{1_A}\cdots(Li^+$ or, $K^+)$ and $\mathbf{1_{B-K}}\cdots Na^+$ complexes. Color code for atoms: H = white; Li = light purple; C = gray; N = blue; O = red; Na = purple; and K = dark purple.

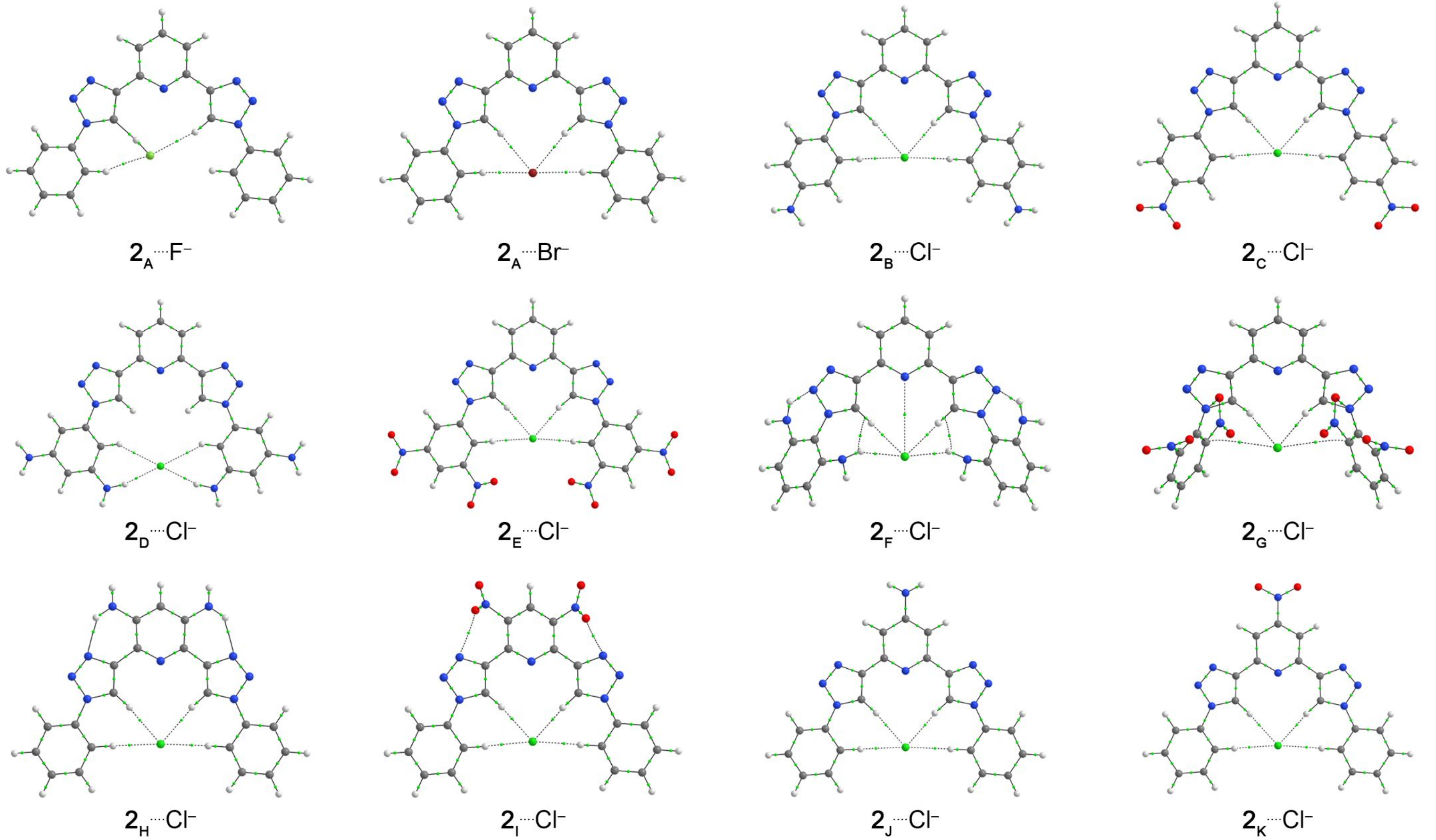

Figure S9. Topological maps showing bond paths (continuous or dashed lines connecting the cores) and bond critical points (small light green points), for the $\mathbf{2_A}$···(F^- or, Br^-) and $\mathbf{2_{B–K}}$···Cl^- complexes. Color code for atoms: H = white; C = gray; N = blue; O = red; F = lemon green; Cl = green; and Br = brown.

Table S1. Ratio of kinetic energy density (G_b) to potential energy density (V_b), expressed as $-G_b/V_b$, along with the electron density (ρ_b) at BCPs related to the interactions in the $\mathbf{1}_{A\text{–}K}\cdots(Li^+$, Na^+ or, $K^+)$ complexes. All values are presented in atomic units (a.u.).

Complex	BCP	$-G_b/V_b$	ρ_b
	N···Li^+	1.177	0.026
$\mathbf{1}_A\cdots Li^+$	N···Li^+	1.243	0.016
	N···Li^+	1.243	0.016
	N···Na^+	1.258	0.022
$\mathbf{1}_A\cdots Na^+$	N···Na^+	1.294	0.019
	N···Na^+	1.294	0.019
	N···K^+	1.198	0.019
$\mathbf{1}_A\cdots K^+$	N···K^+	1.207	0.020
	N···K^+	1.207	0.020
	N···Na^+	1.241	0.024
$\mathbf{1}_B\cdots Na^+$	N···Na^+	1.278	0.020
	N···Na^+	1.278	0.020
	N···Na^+	1.248	0.023
$\mathbf{1}_C\cdots Na^+$	N···Na^+	1.285	0.020
	N···Na^+	1.285	0.020
	N···Na^+	1.244	0.024
$\mathbf{1}_D\cdots Na^+$	N···Na^+	1.278	0.020
	N···Na^+	1.278	0.020
	N···Na^+	1.250	0.023
$\mathbf{1}_E\cdots Na^+$	N···Na^+	1.289	0.020
	N···Na^+	1.289	0.020
	N···Na^+	1.242	0.024
$\mathbf{1}_F\cdots Na^+$	N···Na^+	1.280	0.020
	N···Na^+	1.280	0.020
	N···Na^+	1.256	0.022
$\mathbf{1}_G\cdots Na^+$	N···Na^+	1.306	0.018
	N···Na^+	1.372	0.011
	ONO···Na^+	1.464	0.009
	N···Na^+	1.246	0.023
$\mathbf{1}_H\cdots Na^+$	N···Na^+	1.267	0.022
	N···Na^+	1.267	0.022
	N···Na^+	1.298	0.018
$\mathbf{1}_I\cdots Na^+$	N···Na^+	1.249	0.024
	N···Na^+	1.251	0.024
	N···Na^+	1.233	0.025
$\mathbf{1}_J\cdots Na^+$	N···Na^+	1.283	0.020
	N···Na^+	1.283	0.020
	N···Na^+	1.254	0.023
$\mathbf{1}_K\cdots Na^+$	N···Na^+	1.284	0.020
	N···Na^+	1.284	0.020

Table S2. Ratio of kinetic energy density (G_b) to potential energy density (V_b), expressed as $-G_b/V_b$, along with the electron density (ρ_b) at BCPs related to the interactions in the $\mathbf{2_{A–K}}\cdots(F^-, Cl^-$ or, $Br^-)$ complexes. All values are presented in atomic units (a.u.).

Complex	BCP	$-G_b/V_b$	ρ_b
$\mathbf{2_A}\cdots F^-$	C–H···F⁻	1.347	0.006
	C–H···F⁻	1.366	0.009
	C–H···F⁻	0.228	0.236
$\mathbf{2_A}\cdots Cl^-$	C–H···Cl⁻	1.247	0.015
	C–H···Cl⁻	1.247	0.015
	C–H···Cl⁻	1.211	0.017
	C–H···Cl⁻	1.211	0.017
$\mathbf{2_A}\cdots Br^-$	C–H···Br⁻	1.145	0.016
	C–H···Br⁻	1.120	0.018
	C–H···Br⁻	1.145	0.016
	C–H···Br⁻	1.120	0.018
$\mathbf{2_B}\cdots Cl^-$	C–H···Cl⁻	1.229	0.016
	C–H···Cl⁻	1.210	0.017
	C–H···Cl⁻	1.229	0.016
	C–H···Cl⁻	1.210	0.017
$\mathbf{2_C}\cdots Cl^-$	C–H···Cl⁻	1.172	0.019
	C–H···Cl⁻	1.196	0.018
	C–H···Cl⁻	1.172	0.019
	C–H···Cl⁻	1.196	0.018
$\mathbf{2_D}\cdots Cl^-$	C–H···Cl⁻	1.263	0.011
	C–H···Cl⁻	1.263	0.011
	HN–H···Cl⁻	1.112	0.022
	HN–H···Cl⁻	1.110	0.022
$\mathbf{2_E}\cdots Cl^-$	C–H···Cl⁻	1.136	0.021
	C–H···Cl⁻	1.230	0.016
	C–H···Cl⁻	1.135	0.021
	C–H···Cl⁻	1.232	0.016
$\mathbf{2_F}\cdots Cl^-$	C–H···Cl⁻	1.229	0.016
	C–H···Cl⁻	1.228	0.016
	HN–H···Cl⁻	1.103	0.023
	HN–H···Cl⁻	1.102	0.023
	N···Cl⁻	1.408	0.003
$\mathbf{2_G}\cdots Cl^-$	C–H···Cl⁻	1.151	0.020
	C–H···Cl⁻	1.151	0.020
	O_2N–C···Cl⁻	1.357	0.006
	O_2N–C···Cl⁻	1.358	0.006
$\mathbf{2_H}\cdots Cl^-$	C–H···Cl⁻	1.219	0.017
	C–H···Cl⁻	1.219	0.017
	C–H···Cl⁻	1.192	0.018
	C–H···Cl⁻	1.192	0.018
$\mathbf{2_I}\cdots Cl^-$	C–H···Cl⁻	1.158	0.019
	C–H···Cl⁻	1.158	0.019
	C–H···Cl⁻	1.200	0.018
	C–H···Cl⁻	1.200	0.018
$\mathbf{2_J}\cdots Cl^-$	C–H···Cl⁻	1.217	0.017
	C–H···Cl⁻	1.217	0.017
	C–H···Cl⁻	1.214	0.017
	C–H···Cl⁻	1.214	0.017
$\mathbf{2_K}\cdots Cl^-$	C–H···Cl⁻	1.182	0.018
	C–H···Cl⁻	1.182	0.018
	C–H···Cl⁻	1.223	0.016
	C–H···Cl⁻	1.223	0.016

Table S3. Optimized Cartesian coordinates for the compounds investigated in this study, calculated using the BLYP–D3(BJ)/Def2–TZVP computational model.

1$_{A}$···Li^{+}

C	-1.216213000	2.911303000	0.004972000
C	-1.184121000	1.506315000	0.004433000
N	0.000001000	0.824183000	0.000002000
C	1.184120000	1.506317000	-0.004438000
C	1.216210000	2.911306000	-0.004987000
C	-0.000002000	3.604791000	-0.000009000
C	2.358348000	0.638752000	-0.008363000
N	2.212014000	-0.772262000	-0.014495000
N	3.383992000	-1.329485000	-0.016941000
N	4.346661000	-0.353643000	-0.011978000
C	3.734740000	0.906996000	-0.008047000
C	5.765943000	-0.644126000	-0.011073000
C	6.238431000	-1.759006000	-0.720058000
C	7.611406000	-2.021622000	-0.716649000
C	8.489917000	-1.186492000	-0.019211000
C	8.000486000	-0.081955000	0.688883000
C	6.632748000	0.198902000	0.704107000
N	-3.383994000	-1.329486000	0.016866000
N	-4.346661000	-0.353645000	0.011971000
C	-2.358349000	0.638750000	0.008352000
C	-6.238419000	-1.759015000	0.720063000
C	-7.611393000	-2.021631000	0.716673000
C	-8.489916000	-1.186493000	0.019257000
C	-8.000496000	-0.081949000	-0.688833000
C	-6.632757000	0.198909000	-0.704073000
C	-5.765942000	-0.644126000	0.011084000
H	-2.162882000	3.451029000	0.009783000
H	2.162878000	3.451034000	-0.009805000
H	-0.000003000	4.700302000	-0.000013000
H	4.272323000	1.841710000	-0.017343000
H	5.551696000	-2.415148000	-1.259687000
H	7.996492000	-2.886166000	-1.263196000
H	9.561919000	-1.398104000	-0.023366000
H	8.691062000	0.562571000	1.237895000
H	6.257613000	1.048231000	1.273241000
H	-5.551676000	-2.415164000	1.259672000

H	-7.996472000	-2.886181000	1.263216000
H	-9.561918000	-1.398106000	0.023426000
H	-8.691079000	0.562584000	-1.237828000
H	-6.257631000	1.048244000	-1.273203000
C	-3.734740000	0.906996000	0.008081000
N	-2.212015000	-0.772264000	0.014420000
H	-4.272324000	1.841708000	0.017433000
Li	0.000002000	-1.253287000	-0.000034000

1$_{A}$···Na^{+}

C	-1.215153000	2.859106000	0.005824000
C	-1.182796000	1.451240000	0.005093000
N	-0.000000000	0.773028000	0.000004000
C	1.182796000	1.451239000	-0.005083000
C	1.215153000	2.859105000	-0.005812000
C	0.000000000	3.552508000	0.000006000
C	2.376450000	0.604298000	-0.009449000
N	2.299705000	-0.811044000	-0.021063000
N	3.490962000	-1.315272000	-0.022708000
N	4.414064000	-0.295252000	-0.011161000
C	3.742363000	0.932888000	-0.004207000
C	5.843987000	-0.518413000	-0.007869000
C	6.366903000	-1.625029000	-0.694573000
C	7.750297000	-1.824484000	-0.689828000
C	8.591307000	-0.935417000	-0.013037000
C	8.052964000	0.160046000	0.672799000
C	6.673646000	0.377991000	0.686220000
N	-3.490962000	-1.315272000	0.022723000
N	-4.414064000	-0.295252000	0.011162000
C	-2.376450000	0.604298000	0.009457000
C	-6.366910000	-1.625011000	0.694589000
C	-7.750303000	-1.824466000	0.689836000
C	-8.591306000	-0.935416000	0.013014000
C	-8.052956000	0.160031000	-0.672844000
C	-6.673639000	0.377975000	-0.686258000
C	-5.843987000	-0.518412000	0.007862000
H	-2.160379000	3.399902000	0.011501000
H	2.160378000	3.399902000	-0.011488000
H	0.000000000	4.647203000	0.000008000
H	4.234794000	1.891790000	-0.008475000

H	5.709573000	-2.322698000	-1.218275000
H	8.173395000	-2.681542000	-1.219249000
H	9.671548000	-1.097579000	-0.016271000
H	8.713980000	0.847337000	1.205656000
H	6.261339000	1.221295000	1.238063000
H	-5.709584000	-2.322666000	1.218315000
H	-8.173406000	-2.681511000	1.219274000
H	-9.671548000	-1.097576000	0.016242000
H	-8.713967000	0.847308000	-1.205724000
H	-6.261325000	1.221264000	-1.238118000
C	-3.742364000	0.932888000	0.004204000
N	-2.299705000	-0.811043000	0.021082000
H	-4.234796000	1.891790000	0.008460000
Na	0.000000000	-1.615933000	0.000013000

1$_{A}$···K^{+}

C	-1.210189000	2.758633000	0.006847000
C	-1.177979000	1.348488000	0.005720000
N	-0.000001000	0.659348000	-0.000008000
C	1.177977000	1.348482000	-0.005737000
C	1.210194000	2.758631000	-0.006869000
C	0.000005000	3.456379000	-0.000013000
C	2.407874000	0.556462000	-0.010499000
N	2.431344000	-0.856013000	-0.029601000
N	3.654400000	-1.276967000	-0.030518000
N	4.506113000	-0.195470000	-0.010399000
C	3.750063000	0.981079000	0.000998000
C	5.947526000	-0.318441000	-0.003981000
C	6.547134000	-1.395557000	-0.675082000
C	7.940890000	-1.498829000	-0.667892000
C	8.718062000	-0.544356000	-0.003960000
C	8.104537000	0.520548000	0.666403000
C	6.713373000	0.642574000	0.676989000
N	-3.654402000	-1.276960000	0.030538000
N	-4.506114000	-0.195463000	0.010405000
C	-2.407875000	0.556468000	0.010489000
C	-6.547125000	-1.395565000	0.675089000
C	-7.940880000	-1.498842000	0.667908000
C	-8.718061000	-0.544365000	0.003992000
C	-8.104546000	0.520551000	-0.666360000

C	-6.713382000	0.642584000	-0.676952000
C	-5.947526000	-0.318437000	0.003997000
H	-2.155813000	3.297442000	0.013833000
H	2.155822000	3.297432000	-0.013855000
H	0.000004000	4.550288000	-0.000015000
H	4.172220000	1.971809000	0.003653000
H	5.939627000	-2.144045000	-1.188626000
H	8.422528000	-2.331725000	-1.185327000
H	9.806697000	-0.631327000	-0.005312000
H	8.715724000	1.259445000	1.189250000
H	6.243436000	1.463126000	1.216747000
H	-5.939610000	-2.144057000	1.188618000
H	-8.422512000	-2.331746000	1.185337000
H	-9.806696000	-0.631342000	0.005350000
H	-8.715738000	1.259452000	-1.189194000
H	-6.243454000	1.463149000	-1.216698000
C	-3.750063000	0.981085000	-0.001012000
N	-2.431345000	-0.856006000	0.029612000
H	-4.172221000	1.971816000	-0.003685000
K	-0.000002000	-2.088550000	-0.000038000

$\mathbf{2}_{A}\cdots F^{-}$

C	1.111690000	3.977001000	-0.017043000
C	1.063561000	2.551499000	-0.012775000
N	-0.137419000	1.900286000	-0.012591000
C	-1.290385000	2.634537000	-0.015598000
C	-1.308356000	4.035963000	-0.020630000
C	-0.071644000	4.700266000	-0.021306000
C	-2.497787000	1.808425000	-0.010963000
N	-3.917491000	0.094487000	0.004988000
N	-4.645390000	1.278479000	-0.013024000
C	-4.516923000	-1.210429000	0.019625000
C	-3.690585000	-2.349471000	0.044295000
C	-4.279674000	-3.614360000	0.058361000
C	-5.672035000	-3.751678000	0.048192000
C	-6.484380000	-2.613312000	0.023748000
C	-5.917956000	-1.337834000	0.009280000
N	3.776506000	0.144440000	-0.007645000
N	4.443151000	1.397149000	0.010683000
C	2.253962000	1.737598000	-0.008555000

C	3.859687000	-2.295138000	-0.283767000
C	4.587878000	-3.483747000	-0.281226000
C	5.963009000	-3.479597000	-0.021481000
C	6.612325000	-2.268373000	0.239172000
C	5.902318000	-1.068480000	0.246571000
C	4.515561000	-1.077214000	-0.013189000
H	2.082455000	4.468572000	-0.016672000
H	-2.246523000	4.578980000	-0.023615000
H	-0.048108000	5.788345000	-0.024995000
H	-2.599656000	-2.255823000	0.052648000
H	-3.642795000	-4.498998000	0.077541000
H	-6.120801000	-4.741532000	0.059340000
H	-7.568099000	-2.718220000	0.015862000
H	-6.550548000	-0.448139000	-0.009719000
H	2.787450000	-2.305234000	-0.491238000
H	4.076120000	-4.422446000	-0.485017000
H	6.520974000	-4.409340000	-0.022378000
H	7.681243000	-2.258531000	0.440010000
H	6.409815000	-0.124443000	0.448638000
C	-2.548836000	0.408766000	0.006495000
C	2.399218000	0.340453000	-0.017726000
N	3.561798000	2.320242000	0.007327000
N	-3.832121000	2.280158000	-0.022325000
H	1.262256000	-0.709683000	0.013688000
H	-1.726972000	-0.301109000	0.018600000
F	0.555119000	-1.490924000	0.038505000

$\mathbf{2}_{A}\cdots Cl^{-}$

C	1.212637000	4.223086000	-0.000010000
C	1.175902000	2.813169000	-0.000001000
N	0.000000000	2.122787000	0.000004000
C	-1.175899000	2.813167000	-0.000001000
C	-1.212637000	4.223087000	-0.000010000
C	-0.000001000	4.916681000	-0.000014000
C	-2.359294000	1.963777000	0.000002000
N	-3.758852000	0.239556000	0.000005000
N	-4.497977000	1.417816000	0.000007000
C	-4.364459000	-1.068448000	0.000004000
C	-3.544860000	-2.209314000	-0.000002000
C	-4.141693000	-3.470669000	-0.000002000

C	-5.534741000	-3.600130000	0.000005000
C	-6.341162000	-2.456996000	0.000011000
C	-5.766620000	-1.184851000	0.000011000
N	3.758854000	0.239556000	0.000003000
N	4.497979000	1.417816000	0.000012000
C	2.359295000	1.963777000	0.000002000
C	3.544858000	-2.209314000	-0.000019000
C	4.141689000	-3.470669000	-0.000020000
C	5.534737000	-3.600132000	-0.000001000
C	6.341160000	-2.456999000	0.000021000
C	5.766619000	-1.184853000	0.000023000
C	4.364459000	-1.068448000	0.000002000
H	2.168474000	4.741077000	-0.000013000
H	-2.168476000	4.741073000	-0.000013000
H	0.000001000	6.005757000	-0.000021000
H	-2.438902000	-2.133337000	-0.000007000
H	-3.503078000	-4.355482000	-0.000007000
H	-5.988816000	-4.587167000	0.000005000
H	-7.424414000	-2.555361000	0.000016000
H	-6.394608000	-0.293224000	0.000016000
H	2.438900000	-2.133334000	-0.000033000
H	3.503074000	-4.355482000	-0.000037000
H	5.988810000	-4.587169000	-0.000002000
H	7.424412000	-2.555364000	0.000037000
H	6.394609000	-0.293228000	0.000040000
C	-2.397531000	0.558104000	0.000001000
C	2.397532000	0.558104000	-0.000004000
N	3.694395000	2.428061000	0.000011000
N	-3.694394000	2.428062000	0.000006000
H	1.566659000	-0.165312000	-0.000014000
H	-1.566657000	-0.165311000	-0.000001000
Cl	0.000002000	-2.098617000	-0.000017000

2$_A$···Br^-

C	-1.211733000	4.375334000	-0.000003000
C	-1.175099000	2.965229000	0.000000000
N	0.000001000	2.272695000	0.000003000
C	1.175100000	2.965230000	0.000002000
C	1.211733000	4.375336000	0.000001000
C	0.000000000	5.069836000	-0.000002000

C	2.365543000	2.125364000	0.000002000
N	3.790146000	0.421313000	0.000000000
N	4.512479000	1.609602000	0.000005000
C	4.414770000	-0.877615000	-0.000001000
C	3.612739000	-2.030445000	0.000003000
C	4.227092000	-3.283118000	0.000001000
C	5.621900000	-3.392168000	-0.000005000
C	6.411395000	-2.237255000	-0.000010000
C	5.818520000	-0.973541000	-0.000008000
N	-3.790148000	0.421314000	-0.000001000
N	-4.512478000	1.609604000	0.000000000
C	-2.365542000	2.125362000	0.000000000
C	-3.612741000	-2.030445000	0.000016000
C	-4.227092000	-3.283117000	0.000016000
C	-5.621902000	-3.392167000	0.000001000
C	-6.411397000	-2.237256000	-0.000014000
C	-5.818521000	-0.973541000	-0.000015000
C	-4.414772000	-0.877614000	0.000000000
H	-2.167922000	4.893262000	-0.000005000
H	2.167922000	4.893263000	0.000002000
H	-0.000001000	6.158915000	-0.000002000
H	2.504818000	-1.970149000	0.000009000
H	3.601978000	-4.177480000	0.000005000
H	6.090496000	-4.372472000	-0.000007000
H	7.496094000	-2.319815000	-0.000014000
H	6.433635000	-0.072888000	-0.000011000
H	-2.504819000	-1.970148000	0.000027000
H	-3.601980000	-4.177480000	0.000027000
H	-6.090495000	-4.372472000	0.000001000
H	-7.496095000	-2.319814000	-0.000026000
H	-6.433638000	-0.072889000	-0.000027000
C	2.424216000	0.721381000	-0.000002000
C	-2.424216000	0.721381000	0.000000000
N	-3.694886000	2.608101000	0.000000000
N	3.694887000	2.608101000	0.000006000
H	-1.602736000	-0.013873000	0.000000000
H	1.602732000	-0.013869000	-0.000005000
Br	0.000001000	-2.002407000	0.000001000

1$_B$···Na^+

C	-1.209121000	2.971467000	-0.003562000
C	-1.169396000	1.567428000	-0.002175000
N	0.000000000	0.897454000	0.000002000
C	1.169397000	1.567427000	0.002189000
C	1.209123000	2.971466000	0.003594000
C	0.000001000	3.669699000	0.000021000
C	2.368432000	0.720654000	0.001849000
N	2.270708000	-0.654355000	-0.006400000
N	3.473859000	-1.188740000	-0.003352000
N	4.370556000	-0.156874000	-0.003187000
C	3.718282000	1.040195000	-0.001223000
C	5.777987000	-0.404283000	-0.005320000
C	6.284872000	-1.534919000	-0.660489000
C	7.652412000	-1.774599000	-0.665573000
C	8.548872000	-0.889630000	-0.021653000
C	8.016290000	0.242097000	0.637699000
C	6.646877000	0.478457000	0.649781000
N	-3.473860000	-1.188739000	0.003332000
N	-4.370556000	-0.156872000	0.003176000
C	-2.368432000	0.720655000	-0.001846000
C	-6.284874000	-1.534924000	0.660461000
C	-7.652414000	-1.774602000	0.665541000
C	-8.548873000	-0.889626000	0.021630000
C	-8.016290000	0.242108000	-0.637709000
C	-6.646876000	0.478467000	-0.649786000
C	-5.777987000	-0.404280000	0.005306000
H	-2.156640000	3.503671000	-0.007027000
H	2.156642000	3.503670000	0.007066000
H	0.000001000	4.757550000	0.000028000
H	4.244380000	1.982039000	-0.036302000
H	5.604803000	-2.215980000	-1.163645000
H	8.041387000	-2.649683000	-1.183337000
H	8.684783000	0.929330000	1.153220000
H	6.255724000	1.335630000	1.193201000
H	-5.604805000	-2.215990000	1.163611000
H	-8.041390000	-2.649692000	1.183295000
H	-8.684781000	0.929347000	-1.153224000
H	-6.255722000	1.335647000	-1.193197000
C	-3.718281000	1.040197000	0.001227000

N	-2.270708000	-0.654354000	0.006388000
H	-4.244379000	1.982041000	0.036316000
Na	-0.000000000	-1.450732000	-0.000011000
N	-9.911410000	-1.100853000	0.075937000
H	-10.506852000	-0.593543000	-0.567343000
H	-10.248937000	-2.019373000	0.337043000
N	9.911410000	-1.100857000	-0.075964000
H	10.248935000	-2.019375000	-0.337078000
H	10.506851000	-0.593556000	0.567323000

1$_{C}$···Na^{+}

C	-1.207996000	3.082745000	-0.393473000
C	-1.167589000	1.691009000	-0.214629000
N	-0.000001000	1.022645000	-0.128770000
C	1.167591000	1.691004000	-0.214614000
C	1.208006000	3.082740000	-0.393457000
C	0.000007000	3.776807000	-0.482901000
C	2.370234000	0.854191000	-0.106796000
N	2.276053000	-0.514559000	0.053561000
N	3.473969000	-1.042633000	0.121342000
N	4.369815000	-0.015528000	0.004475000
C	3.717332000	1.174373000	-0.138788000
C	5.778202000	-0.267872000	0.044260000
C	6.263261000	-1.496987000	-0.421103000
C	7.633992000	-1.744069000	-0.382762000
C	8.486994000	-0.755823000	0.109876000
C	8.012274000	0.469690000	0.576532000
C	6.640330000	0.713945000	0.549894000
N	-3.473978000	-1.042621000	0.121295000
N	-4.369821000	-0.015513000	0.004420000
C	-2.370236000	0.854201000	-0.106822000
C	-6.263270000	-1.496963000	-0.421175000
C	-7.633998000	-1.744053000	-0.382815000
C	-8.486996000	-0.755818000	0.109855000
C	-8.012274000	0.469691000	0.576520000
C	-6.640332000	0.713952000	0.549861000
C	-5.778207000	-0.267858000	0.044208000
H	-2.155500000	3.610547000	-0.461343000
H	2.155513000	3.610538000	-0.461313000
H	0.000010000	4.855457000	-0.622280000

H	4.241509000	2.107866000	-0.277111000
H	5.574175000	-2.240893000	-0.807847000
H	8.049059000	-2.682534000	-0.736414000
H	8.711457000	1.204062000	0.963459000
H	6.249381000	1.648255000	0.943836000
H	-5.574189000	-2.240853000	-0.807959000
H	-8.049070000	-2.682507000	-0.736491000
H	-8.711455000	1.204060000	0.963453000
H	-6.249382000	1.648267000	0.943791000
C	-3.717333000	1.174391000	-0.138795000
N	-2.276061000	-0.514550000	0.053528000
H	-4.241506000	2.107889000	-0.277105000
Na	-0.000006000	-1.321029000	0.173212000
N	9.960004000	-1.019434000	0.142483000
O	10.686203000	-0.116703000	0.578090000
O	10.346427000	-2.120544000	-0.268739000
N	-9.960001000	-1.019451000	0.142520000
O	-10.346415000	-2.120604000	-0.268595000
O	-10.686184000	-0.116778000	0.578271000

1$_D$···Na^+

C	1.209069000	2.898398000	-0.799728000
C	1.169025000	1.549268000	-0.411899000
N	-0.000000000	0.905045000	-0.225646000
C	-1.169025000	1.549268000	-0.411899000
C	-1.209067000	2.898398000	-0.799728000
C	0.000001000	3.569724000	-0.992607000
C	-2.367832000	0.734108000	-0.181629000
N	-2.270331000	-0.592389000	0.185672000
N	-3.472778000	-1.106691000	0.326774000
N	-4.366823000	-0.114430000	0.047106000
C	-3.717015000	1.039136000	-0.272491000
C	-5.783416000	-0.348865000	0.099085000
C	-6.263918000	-1.596129000	-0.291429000
C	-7.657084000	-1.819281000	-0.251736000
C	-8.511097000	-0.784451000	0.168091000
C	-8.005733000	0.467600000	0.557055000
C	-6.612275000	0.689422000	0.528381000
N	3.472778000	-1.106693000	0.326774000
N	4.366823000	-0.114432000	0.047106000

C	2.367832000	0.734107000	-0.181629000
C	6.263917000	-1.596132000	-0.291429000
C	7.657083000	-1.819284000	-0.251735000
C	8.511096000	-0.784455000	0.168091000
C	8.005733000	0.467596000	0.557055000
C	6.612275000	0.689419000	0.528381000
C	5.783416000	-0.348868000	0.099085000
H	2.156915000	3.408975000	-0.947626000
H	-2.156914000	3.408976000	-0.947626000
H	0.000001000	4.615028000	-1.293804000
H	-4.247598000	1.933862000	-0.560371000
H	-5.579750000	-2.367189000	-0.630979000
H	-9.587312000	-0.954542000	0.187326000
H	-6.197205000	1.634436000	0.868544000
H	5.579748000	-2.367191000	-0.630978000
H	9.587311000	-0.954546000	0.187326000
H	6.197205000	1.634433000	0.868544000
C	3.717015000	1.039134000	-0.272491000
N	2.270330000	-0.592390000	0.185672000
H	4.247599000	1.933860000	-0.560371000
Na	-0.000000000	-1.358107000	0.421531000
N	-8.178052000	-3.029550000	-0.689270000
H	-7.554180000	-3.828309000	-0.692577000
H	-9.126739000	-3.253455000	-0.411179000
N	-8.869049000	1.494040000	0.923435000
H	-8.481127000	2.245380000	1.482765000
H	-9.805599000	1.226320000	1.205322000
N	8.178050000	-3.029554000	-0.689269000
H	9.126737000	-3.253459000	-0.411178000
H	7.554178000	-3.828313000	-0.692576000
N	8.869049000	1.494036000	0.923435000
H	9.805599000	1.226316000	1.205322000
H	8.481128000	2.245376000	1.482766000

1$_{E}$···Na^{+}

C	1.207111000	-3.194078000	0.003761000
C	1.166628000	-1.791115000	0.004451000
N	-0.000192000	-1.115734000	0.001785000
C	-1.167261000	-1.790687000	-0.000420000
C	-1.208259000	-3.193634000	0.001035000

C	-0.000703000	-3.894054000	0.002554000
C	-2.371193000	-0.948031000	-0.005088000
N	-2.278122000	0.430571000	-0.014643000
N	-3.474606000	0.962736000	-0.022685000
N	-4.369450000	-0.072284000	-0.019817000
C	-3.717582000	-1.271738000	-0.008274000
C	-5.775720000	0.187579000	-0.023457000
C	-6.248841000	1.367780000	-0.606779000
C	-7.621243000	1.598235000	-0.592288000
C	-8.533395000	0.704169000	-0.036041000
C	-8.014117000	-0.457040000	0.528271000
C	-6.649444000	-0.734791000	0.557483000
N	3.474993000	0.961446000	0.027958000
N	4.369462000	-0.073887000	0.022253000
C	2.370872000	-0.948899000	0.008894000
C	6.250246000	1.364563000	0.608733000
C	7.622701000	1.594595000	0.592490000
C	8.533709000	0.701095000	0.033462000
C	8.013173000	-0.459044000	-0.531889000
C	6.648364000	-0.736288000	-0.559468000
C	5.775832000	0.185458000	0.024247000
H	2.154292000	-3.726862000	0.004461000
H	-2.155636000	-3.726071000	0.000838000
H	-0.000903000	-4.981612000	0.002881000
H	-4.240425000	-2.216221000	-0.030166000
H	-5.578409000	2.088907000	-1.060562000
H	-9.599439000	0.904668000	-0.042302000
H	-6.306738000	-1.641878000	1.044175000
H	5.580748000	2.085225000	1.064633000
H	9.599822000	0.901260000	0.038354000
H	6.304634000	-1.642476000	-1.047114000
C	3.717148000	-1.273091000	0.009981000
N	2.278308000	0.429722000	0.020442000
H	4.239682000	-2.217791000	0.029749000
Na	0.000249000	1.252447000	0.003503000
N	-8.137272000	2.863847000	-1.218711000
O	-9.357316000	3.044829000	-1.190545000
O	-7.297369000	3.626397000	-1.711693000
N	-8.952884000	-1.452433000	1.146460000
O	-10.153734000	-1.173756000	1.136253000

O	-8.446734000	-2.481601000	1.614628000
N	8.140101000	2.859116000	1.219991000
O	7.301403000	3.620144000	1.717360000
O	9.360358000	3.038704000	1.192161000
N	8.950648000	-1.453746000	-1.153148000
O	8.443036000	-2.480224000	-1.625620000
O	10.151224000	-1.173754000	-1.147716000

1$_{F}$···Na^+

C	-1.209107000	2.678507000	-1.072029000
C	-1.168666000	1.386552000	-0.523943000
N	-0.000000000	0.769316000	-0.260413000
C	1.168666000	1.386551000	-0.523943000
C	1.209108000	2.678506000	-1.072029000
C	0.000001000	3.321597000	-1.344672000
C	2.369435000	0.606229000	-0.198250000
N	2.274708000	-0.665124000	0.330563000
N	3.479573000	-1.152004000	0.537649000
N	4.375084000	-0.198185000	0.131627000
C	3.716721000	0.899540000	-0.334093000
C	5.794223000	-0.415944000	0.166091000
C	6.607852000	0.508002000	0.861010000
C	8.001330000	0.355888000	0.786753000
C	8.548530000	-0.696939000	0.052039000
C	7.741925000	-1.625877000	-0.602103000
C	6.340949000	-1.506489000	-0.552879000
N	-3.479576000	-1.152001000	0.537646000
N	-4.375085000	-0.198181000	0.131624000
C	-2.369435000	0.606231000	-0.198251000
C	-6.607854000	0.508009000	0.861004000
C	-8.001332000	0.355895000	0.786747000
C	-8.548532000	-0.696933000	0.052034000
C	-7.741927000	-1.625871000	-0.602107000
C	-6.340951000	-1.506485000	-0.552882000
C	-5.794225000	-0.415939000	0.166087000
H	-2.156953000	3.166726000	-1.282335000
H	2.156955000	3.166723000	-1.282336000
H	0.000002000	4.322434000	-1.770803000
H	4.251183000	1.763373000	-0.700337000
H	8.644763000	1.055092000	1.316659000

H	9.630025000	-0.807200000	0.007011000
H	8.185478000	-2.454113000	-1.150873000
H	-8.644764000	1.055101000	1.316651000
H	-9.630027000	-0.807193000	0.007004000
H	-8.185480000	-2.454109000	-1.150876000
C	-3.716721000	0.899544000	-0.334092000
N	-2.274711000	-0.665122000	0.330563000
H	-4.251182000	1.763378000	-0.700335000
Na	-0.000002000	-1.399559000	0.650014000
N	6.038464000	1.590705000	1.546257000
H	5.151532000	1.405978000	2.007340000
H	6.684775000	2.067642000	2.168404000
N	-6.038465000	1.590712000	1.546250000
H	-6.684778000	2.067652000	2.168394000
H	-5.151536000	1.405983000	2.007338000
N	5.518412000	-2.382750000	-1.254631000
H	4.621692000	-2.592351000	-0.826894000
H	5.986534000	-3.206355000	-1.618181000
N	-5.518415000	-2.382748000	-1.254631000
H	-5.986537000	-3.206353000	-1.618181000
H	-4.621695000	-2.592348000	-0.826895000

1$_{G}$···Na^{+}

C	-1.271073000	-3.479233000	1.156827000
C	-1.303088000	-2.290157000	0.421788000
N	-0.191384000	-1.576626000	0.156834000
C	1.005830000	-2.011023000	0.594343000
C	1.126657000	-3.199572000	1.332823000
C	-0.028010000	-3.931163000	1.614663000
C	2.132784000	-1.135449000	0.252650000
N	1.934098000	0.027278000	-0.470734000
N	3.066401000	0.650779000	-0.643996000
N	4.033342000	-0.120503000	-0.036479000
C	3.482989000	-1.225435000	0.535016000
C	5.363934000	0.389108000	0.043746000
C	5.622394000	1.546323000	0.795088000
C	6.876888000	2.153466000	0.804463000
C	7.914770000	1.592061000	0.058809000
C	7.684156000	0.457101000	-0.719365000
C	6.419774000	-0.132450000	-0.719953000

N	-3.531729000	-0.349714000	-1.576870000
N	-4.297100000	-0.438410000	-0.424812000
C	-2.512235000	-1.646693000	-0.122720000
C	-4.787652000	1.945762000	-0.345616000
C	-5.663981000	3.028029000	-0.271163000
C	-7.016933000	2.794501000	-0.021585000
C	-7.489622000	1.488070000	0.107430000
C	-6.600337000	0.416396000	0.016125000
C	-5.229680000	0.614180000	-0.205337000
H	-2.182028000	-4.040762000	1.346685000
H	2.097187000	-3.543192000	1.681141000
H	0.039853000	-4.855878000	2.183252000
H	4.076953000	-1.951598000	1.066830000
H	7.022020000	3.054969000	1.392607000
H	8.900959000	2.048708000	0.072166000
H	8.464917000	0.026191000	-1.339529000
H	-5.278589000	4.034811000	-0.403786000
H	-7.705965000	3.631150000	0.057303000
H	-8.544822000	1.280152000	0.260466000
C	-3.678981000	-1.248120000	0.491063000
N	-2.470887000	-1.075111000	-1.383851000
H	-4.087501000	-1.426261000	1.473314000
Na	-0.412344000	0.493513000	-1.017980000
N	6.220906000	-1.327666000	-1.586389000
O	6.733625000	-1.292270000	-2.707061000
O	5.576878000	-2.273705000	-1.107098000
N	4.549428000	2.161941000	1.632493000
O	4.498041000	3.393881000	1.647090000
O	3.817525000	1.392148000	2.270736000
N	-7.164629000	-0.957719000	0.130595000
O	-6.500927000	-1.787424000	0.770277000
O	-8.258414000	-1.154411000	-0.401554000
N	-3.350498000	2.256790000	-0.593176000
O	-3.097703000	3.134363000	-1.415884000
O	-2.490413000	1.630698000	0.067321000

$\mathbf{1}_{H}\cdots Na^{+}$

C	-1.207236000	2.678804000	-0.194155000
C	-1.172943000	1.256683000	-0.112455000
N	0.000000000	0.613914000	0.000001000

C	1.172943000	1.256684000	0.112455000
C	1.207236000	2.678805000	0.194151000
C	0.000000000	3.362088000	-0.000004000
C	2.362425000	0.396542000	0.135395000
N	2.234374000	-0.976827000	0.254658000
N	3.416611000	-1.549348000	0.199497000
N	4.334004000	-0.557925000	0.044591000
C	3.719528000	0.662328000	-0.001880000
C	5.733332000	-0.855903000	-0.051410000
C	6.249095000	-1.946932000	0.656995000
C	7.612355000	-2.232338000	0.559474000
C	8.449817000	-1.432612000	-0.226899000
C	7.919735000	-0.345199000	-0.929590000
C	6.555667000	-0.054058000	-0.851346000
N	-3.416610000	-1.549349000	-0.199486000
N	-4.334004000	-0.557925000	-0.044584000
C	-2.362425000	0.396542000	-0.135392000
C	-6.249095000	-1.946934000	-0.656986000
C	-7.612354000	-2.232339000	-0.559465000
C	-8.449816000	-1.432612000	0.226906000
C	-7.919735000	-0.345197000	0.929595000
C	-6.555666000	-0.054056000	0.851351000
C	-5.733332000	-0.855904000	0.051417000
H	0.000000000	4.452287000	-0.000006000
H	4.276096000	1.573073000	-0.152980000
H	5.589147000	-2.552158000	1.271135000
H	8.021812000	-3.077837000	1.107556000
H	9.511620000	-1.658043000	-0.294541000
H	8.564186000	0.271226000	-1.552094000
H	6.135837000	0.768117000	-1.425907000
H	-5.589146000	-2.552162000	-1.271124000
H	-8.021811000	-3.077840000	-1.107544000
H	-9.511620000	-1.658043000	0.294549000
H	-8.564185000	0.271229000	1.552097000
H	-6.135837000	0.768120000	1.425910000
C	-3.719527000	0.662327000	0.001883000
N	-2.234373000	-0.976828000	-0.254648000
H	-4.276096000	1.573073000	0.152979000
Na	0.000000000	-1.751446000	0.000007000
N	-2.396727000	3.371515000	-0.391377000

H	-3.080020000	2.921294000	-0.993121000
H	-2.295443000	4.359514000	-0.605971000
N	2.396727000	3.371516000	0.391370000
H	3.080021000	2.921297000	0.993115000
H	2.295443000	4.359516000	0.605960000

1$_{I}$···Na^+

C	-1.210924000	2.219251000	-0.374004000
C	-1.193260000	0.806726000	-0.338609000
N	-0.000945000	0.175375000	-0.303541000
C	1.181670000	0.823715000	-0.354296000
C	1.178411000	2.236767000	-0.405000000
C	-0.021393000	2.932955000	-0.365946000
C	2.363257000	-0.045150000	-0.272661000
N	2.194388000	-1.412937000	-0.153018000
N	3.357788000	-2.006498000	-0.066877000
N	4.310501000	-1.025473000	-0.119094000
C	3.732492000	0.196152000	-0.244845000
C	5.708347000	-1.347015000	-0.061335000
C	6.124457000	-2.443578000	0.701451000
C	7.486101000	-2.747090000	0.754918000
C	8.414661000	-1.961080000	0.062508000
C	7.981240000	-0.868493000	-0.696747000
C	6.621814000	-0.557197000	-0.769036000
N	-3.331433000	-2.052501000	-0.056317000
N	-4.297312000	-1.085330000	-0.116568000
C	-2.362880000	-0.078085000	-0.258079000
C	-6.120801000	-2.642394000	-0.591342000
C	-7.478111000	-2.962973000	-0.528825000
C	-8.388935000	-2.076449000	0.057570000
C	-7.941530000	-0.864203000	0.594516000
C	-6.585695000	-0.533103000	0.549402000
C	-5.690377000	-1.425668000	-0.051007000
H	-0.027744000	4.019310000	-0.342432000
H	4.316339000	1.100044000	-0.294604000
H	5.394247000	-3.037975000	1.242350000
H	7.821955000	-3.595458000	1.346594000
H	9.474149000	-2.201360000	0.112063000
H	8.698798000	-0.263095000	-1.245369000
H	6.279378000	0.270650000	-1.385324000

H	-5.404557000	-3.314690000	-1.053915000
H	-7.824931000	-3.904522000	-0.948082000
H	-9.445468000	-2.330920000	0.098585000
H	-8.644907000	-0.178605000	1.060991000
H	-6.231390000	0.392861000	0.995779000
C	-3.735903000	0.144160000	-0.245734000
N	-2.175808000	-1.443835000	-0.141740000
H	-4.332212000	1.033874000	-0.362642000
Na	0.017426000	-2.278228000	-0.130337000
N	2.410814000	3.059086000	-0.491574000
O	2.513913000	3.998403000	0.301721000
O	3.226816000	2.753421000	-1.375108000
N	-2.458887000	3.021537000	-0.406727000
O	-3.270266000	2.758204000	-1.307158000
O	-2.577962000	3.902846000	0.448759000

1$_J$···Na$^+$

C	-1.210942000	2.650742000	-0.198837000
C	-1.162509000	1.261678000	-0.091565000
N	0.000000000	0.575826000	-0.038956000
C	1.162509000	1.261678000	-0.091564000
C	1.210942000	2.650743000	-0.198836000
C	-0.000000000	3.378884000	-0.255226000
C	2.369512000	0.422865000	-0.027426000
N	2.277523000	-0.951847000	0.055701000
N	3.479234000	-1.481148000	0.099052000
N	4.371367000	-0.448988000	0.041361000
C	3.717031000	0.744819000	-0.037956000
C	5.783766000	-0.694485000	0.069223000
C	6.287113000	-1.854574000	-0.529743000
C	7.663460000	-2.087361000	-0.499416000
C	8.524613000	-1.169051000	0.112418000
C	8.006481000	-0.013928000	0.707796000
C	6.630571000	0.227036000	0.695773000
N	-3.479233000	-1.481149000	0.099049000
N	-4.371366000	-0.448989000	0.041355000
C	-2.369511000	0.422864000	-0.027428000
C	-6.287110000	-1.854578000	-0.529750000
C	-7.663457000	-2.087365000	-0.499426000
C	-8.524612000	-1.169055000	0.112405000

C	-8.006482000	-0.013931000	0.707783000
C	-6.630573000	0.227034000	0.695763000
C	-5.783765000	-0.694488000	0.069215000
H	-2.163010000	3.175219000	-0.238638000
H	2.163009000	3.175219000	-0.238637000
H	4.243366000	1.683414000	-0.122189000
H	5.608068000	-2.553132000	-1.009274000
H	8.063777000	-2.985917000	-0.963332000
H	9.596065000	-1.354921000	0.128577000
H	8.670563000	0.695662000	1.195910000
H	6.222263000	1.104895000	1.190912000
H	-5.608064000	-2.553136000	-1.009279000
H	-8.063773000	-2.985922000	-0.963341000
H	-9.596064000	-1.354925000	0.128562000
H	-8.670565000	0.695659000	1.195895000
H	-6.222266000	1.104893000	1.190901000
C	-3.717031000	0.744817000	-0.037961000
N	-2.277522000	-0.951848000	0.055701000
H	-4.243366000	1.683412000	-0.122197000
Na	0.000001000	-1.748990000	0.128867000
N	-0.000001000	4.742811000	-0.335558000
H	-0.861537000	5.248093000	-0.495208000
H	0.861536000	5.248094000	-0.495208000

1$_{K}$···Na^{+}

C	-1.217363000	2.322680000	-0.002462000
C	-1.170580000	0.918521000	-0.001204000
N	0.000000000	0.249827000	-0.000061000
C	1.170582000	0.918520000	0.001097000
C	1.217366000	2.322679000	0.002383000
C	0.000002000	2.992677000	-0.000028000
C	2.371205000	0.077695000	0.000572000
N	2.276371000	-1.298602000	-0.009469000
N	3.477682000	-1.828168000	-0.009251000
N	4.371382000	-0.793470000	-0.003801000
C	3.719330000	0.402061000	0.001400000
C	5.785093000	-1.040965000	-0.002663000
C	6.282363000	-2.157238000	-0.684033000
C	7.659028000	-2.389600000	-0.680792000
C	8.523980000	-1.514218000	-0.013809000

C	8.010749000	-0.402938000	0.663869000
C	6.634970000	-0.162642000	0.679182000
N	-3.477683000	-1.828163000	0.009152000
N	-4.371383000	-0.793465000	0.003748000
C	-2.371204000	0.077698000	-0.000663000
C	-6.282329000	-2.157265000	0.684010000
C	-7.658994000	-2.389625000	0.680834000
C	-8.523981000	-1.514215000	0.013934000
C	-8.010785000	-0.402908000	-0.663724000
C	-6.635007000	-0.162610000	-0.679098000
C	-5.785094000	-1.040962000	0.002664000
H	-2.146534000	2.882120000	-0.004796000
H	2.146538000	2.882117000	0.004781000
H	4.244220000	1.345307000	-0.024640000
H	5.600262000	-2.821563000	-1.206029000
H	8.056445000	-3.253192000	-1.208977000
H	9.595617000	-1.699427000	-0.019224000
H	8.678420000	0.272287000	1.193794000
H	6.230541000	0.679770000	1.235366000
H	-5.600201000	-2.821610000	1.205946000
H	-8.056383000	-3.253237000	1.209007000
H	-9.595618000	-1.699424000	0.019398000
H	-8.678484000	0.272341000	-1.193585000
H	-6.230610000	0.679827000	-1.235266000
C	-3.719329000	0.402065000	-0.001454000
N	-2.276371000	-1.298599000	0.009345000
H	-4.244217000	1.345311000	0.024637000
Na	-0.000001000	-2.121171000	-0.000105000
N	0.000002000	4.499652000	0.000064000
O	1.101130000	5.058862000	0.011515000
O	-1.101120000	5.058865000	-0.011647000

$\mathbf{2}_{B}\cdots Cl^{-}$

C	-1.210490000	4.548180000	0.017132000
C	-1.162671000	3.140618000	0.012470000
N	0.000000000	2.464299000	0.011144000
C	1.162672000	3.140617000	0.012469000
C	1.210492000	4.548179000	0.017135000
C	0.000001000	5.245636000	0.019820000
C	2.357670000	2.295995000	0.007933000

N	3.711454000	0.598158000	-0.002435000
N	4.480813000	1.754786000	0.005954000
C	4.322746000	-0.691547000	-0.008308000
C	3.527580000	-1.844359000	-0.089482000
C	4.137556000	-3.097063000	-0.084299000
C	5.534217000	-3.230937000	0.001681000
C	6.315135000	-2.063310000	0.077613000
C	5.717673000	-0.803437000	0.075705000
N	-3.711455000	0.598161000	-0.002441000
N	-4.480813000	1.754789000	0.005961000
C	-2.357670000	2.295997000	0.007929000
C	-3.527582000	-1.844356000	-0.089488000
C	-4.137557000	-3.097061000	-0.084304000
C	-5.534218000	-3.230934000	0.001679000
C	-6.315135000	-2.063307000	0.077615000
C	-5.717674000	-0.803434000	0.075707000
C	-4.322746000	-0.691544000	-0.008312000
H	-2.170639000	5.057745000	0.018150000
H	2.170642000	5.057743000	0.018155000
H	0.000002000	6.335617000	0.023269000
H	2.435718000	-1.788061000	-0.146137000
H	3.507149000	-3.984484000	-0.144873000
H	7.400997000	-2.143017000	0.139337000
H	6.320873000	0.096704000	0.140431000
H	-2.435719000	-1.788058000	-0.146144000
H	-3.507151000	-3.984481000	-0.144879000
H	-7.400997000	-2.143014000	0.139342000
H	-6.320873000	0.096707000	0.140436000
C	2.386846000	0.906828000	-0.001936000
C	-2.386847000	0.906830000	-0.001942000
N	-3.649023000	2.768729000	0.012445000
N	3.649023000	2.768726000	0.012479000
H	-1.582458000	0.168806000	-0.007057000
H	1.582457000	0.168804000	-0.007051000
Cl	-0.000001000	-1.708599000	-0.076198000
N	6.144608000	-4.505277000	-0.053943000
H	5.535272000	-5.260334000	0.250727000
H	7.041926000	-4.552555000	0.422197000
N	-6.144609000	-4.505274000	-0.053944000
H	-7.041926000	-4.552552000	0.422199000

H	-5.535273000	-5.260331000	0.250724000

2$_C$···Cl⁻

C	1.210517000	5.109812000	-0.000069000
C	1.161272000	3.703172000	-0.000014000
N	0.000004000	3.026743000	-0.000003000
C	-1.161263000	3.703175000	0.000014000
C	-1.210505000	5.109815000	0.000090000
C	0.000007000	5.807596000	0.000015000
C	-2.351507000	2.851594000	0.000094000
N	-3.696060000	1.147186000	0.000095000
N	-4.475961000	2.308842000	-0.000123000
C	-4.301481000	-0.133312000	0.000071000
C	-3.497696000	-1.287544000	0.000059000
C	-4.109905000	-2.538158000	0.000009000
C	-5.506043000	-2.626938000	-0.000026000
C	-6.309410000	-1.480036000	-0.000028000
C	-5.706108000	-0.228689000	0.000007000
N	3.696062000	1.147176000	-0.000102000
N	4.475966000	2.308830000	0.000094000
C	2.351514000	2.851587000	-0.000094000
C	3.497692000	-1.287554000	-0.000039000
C	4.109897000	-2.538169000	0.000009000
C	5.506036000	-2.626952000	0.000005000
C	6.309405000	-1.480053000	-0.000026000
C	5.706106000	-0.228704000	-0.000059000
C	4.301480000	-0.133323000	-0.000084000
H	2.169986000	5.620445000	-0.000126000
H	-2.169972000	5.620451000	0.000159000
H	0.000009000	6.897029000	0.000024000
H	-2.403965000	-1.227157000	0.000046000
H	-3.508560000	-3.441606000	-0.000006000
H	-7.389414000	-1.583960000	-0.000066000
H	-6.300640000	0.678508000	-0.000004000
H	2.403961000	-1.227163000	0.000001000
H	3.508551000	-3.441616000	0.000052000
H	7.389409000	-1.583980000	-0.000016000
H	6.300641000	0.678492000	-0.000075000
C	-2.370225000	1.466183000	0.000101000
C	2.370228000	1.466177000	-0.000102000

N	3.650524000	3.317277000	-0.000462000
N	-3.650516000	3.317286000	0.000502000
H	1.558620000	0.733127000	-0.000076000
H	-1.558619000	0.733131000	0.000074000
Cl	-0.000002000	-1.060731000	0.000013000
N	-6.152514000	-3.953514000	-0.000063000
O	-7.399553000	-3.997631000	-0.000214000
O	-5.423887000	-4.962273000	-0.000166000
N	6.152503000	-3.953531000	0.000056000
O	5.423874000	-4.962288000	0.000253000
O	7.399542000	-3.997651000	0.000206000

$\mathbf{2}_D\cdots Cl^-$

C	-1.211250000	4.832091000	-0.062320000
C	-1.164673000	3.425673000	-0.043861000
N	-0.000719000	2.748847000	-0.031678000
C	1.163402000	3.425289000	-0.043286000
C	1.210462000	4.831739000	-0.061640000
C	-0.000273000	5.528981000	-0.070065000
C	2.354717000	2.574927000	-0.044586000
N	3.682074000	0.850736000	-0.042526000
N	4.467756000	1.988981000	-0.089037000
C	4.237855000	-0.469086000	-0.030042000
C	3.389329000	-1.537335000	-0.303475000
C	3.891992000	-2.854869000	-0.235563000
C	5.253902000	-3.048689000	0.077443000
C	6.096348000	-1.954310000	0.325114000
C	5.592565000	-0.643810000	0.281383000
N	-3.683071000	0.851040000	-0.043876000
N	-4.468978000	1.989024000	-0.092008000
C	-2.356136000	2.575511000	-0.045689000
C	-3.389018000	-1.536741000	-0.304627000
C	-3.890519000	-2.854732000	-0.235928000
C	-5.252274000	-3.049364000	0.077466000
C	-6.095470000	-1.955512000	0.324899000
C	-5.592683000	-0.644617000	0.280753000
C	-4.238197000	-0.469061000	-0.031130000
H	-2.171254000	5.341639000	-0.072717000
H	2.170645000	5.340950000	-0.071474000
H	-0.000111000	6.618563000	-0.084538000

H	2.336838000	-1.405601000	-0.539271000
H	5.657166000	-4.061533000	0.129050000
H	6.221401000	0.215184000	0.493476000
H	-2.336755000	-1.404171000	-0.540923000
H	-5.654774000	-4.062489000	0.129699000
H	-6.221983000	0.213993000	0.493030000
C	2.368235000	1.189450000	-0.012773000
C	-2.369369000	1.190049000	-0.012905000
N	-3.656420000	3.022162000	-0.091590000
N	3.655000000	3.021892000	-0.088825000
H	-1.566170000	0.470965000	0.041778000
H	1.565071000	0.470178000	0.040534000
Cl	0.000574000	-2.655509000	-0.296911000
N	3.042909000	-3.907690000	-0.530918000
H	2.033095000	-3.687594000	-0.480469000
H	3.296133000	-4.808571000	-0.139874000
N	7.447207000	-2.172170000	0.696212000
H	8.050869000	-1.382162000	0.481436000
H	7.836027000	-3.029806000	0.311560000
N	-3.040318000	-3.906548000	-0.530688000
H	-3.293252000	-4.808334000	-0.141659000
H	-2.030644000	-3.685338000	-0.479969000
N	-7.446084000	-2.174211000	0.696369000
H	-7.833731000	-3.033122000	0.313401000
H	-8.050768000	-1.385381000	0.480208000

2$_E$···Cl^-

C	-1.263728000	5.403452000	-0.014091000
C	-1.199235000	3.998328000	0.004859000
N	-0.031474000	3.336434000	-0.045106000
C	1.122132000	4.020986000	-0.113759000
C	1.157201000	5.427181000	-0.134563000
C	-0.060559000	6.111868000	-0.084233000
C	2.307846000	3.163690000	-0.164583000
N	3.614241000	1.434279000	-0.210853000
N	4.420231000	2.577832000	-0.229495000
C	4.189081000	0.139924000	-0.234542000
C	3.405511000	-0.974671000	-0.582516000
C	4.022518000	-2.225437000	-0.568437000
C	5.359028000	-2.419152000	-0.232716000

C	6.100168000	-1.280376000	0.078265000
C	5.544558000	-0.001953000	0.088558000
N	-3.634598000	1.362486000	0.179222000
N	-4.465648000	2.487829000	0.160656000
C	-2.366456000	3.117824000	0.081173000
C	-3.374882000	-1.026770000	0.634467000
C	-3.962544000	-2.291454000	0.656493000
C	-5.290445000	-2.527605000	0.313794000
C	-6.054268000	-1.417707000	-0.043285000
C	-5.528257000	-0.127549000	-0.090938000
C	-4.180176000	0.056790000	0.241079000
H	-2.226326000	5.906702000	0.026438000
H	2.109142000	5.948977000	-0.189370000
H	-0.071982000	7.201000000	-0.099611000
H	2.338139000	-0.896390000	-0.809474000
H	5.802689000	-3.406956000	-0.220576000
H	6.146153000	0.862542000	0.341555000
H	-2.312196000	-0.915793000	0.868783000
H	-5.710689000	-3.525518000	0.330797000
H	-6.146865000	0.713573000	-0.379312000
C	2.298690000	1.777283000	-0.172428000
C	-2.326847000	1.732920000	0.133036000
N	-3.681581000	3.529207000	0.104554000
N	3.613573000	3.602876000	-0.203768000
H	-1.493500000	1.025260000	0.112709000
H	1.480962000	1.052792000	-0.127334000
N	3.225398000	-3.421396000	-0.974434000
O	3.622578000	-4.529536000	-0.571960000
O	2.241660000	-3.228293000	-1.693632000
N	7.539799000	-1.436823000	0.419626000
O	8.020021000	-2.582086000	0.384489000
O	8.178191000	-0.415094000	0.719715000
N	-3.142369000	-3.453736000	1.111159000
O	-2.170473000	-3.213000000	1.832154000
O	-3.510117000	-4.584152000	0.744299000
N	-7.485819000	-1.619319000	-0.394563000
O	-8.143795000	-0.623724000	-0.737289000
O	-7.940290000	-2.773444000	-0.324282000
Cl	0.012335000	-0.751651000	0.025328000

2$_{F}$···Cl^{-}

C	-1.208568000	4.237269000	-0.225688000
C	-1.160834000	2.839796000	-0.058387000
N	0.000062000	2.165982000	0.037757000
C	1.160926000	2.839735000	-0.059059000
C	1.208679000	4.237190000	-0.226476000
C	0.000050000	4.931891000	-0.302606000
C	2.366907000	2.012503000	-0.002327000
N	3.759844000	0.340382000	0.081226000
N	4.498224000	1.517559000	-0.051153000
C	4.432416000	-0.922177000	0.165285000
C	4.068270000	-1.986653000	-0.700072000
C	4.806369000	-3.190306000	-0.610968000
C	5.849926000	-3.322968000	0.298615000
C	6.197357000	-2.275253000	1.152627000
C	5.493971000	-1.058201000	1.094021000
N	-3.759676000	0.340428000	0.082038000
N	-4.498208000	1.517842000	-0.048131000
C	-2.366815000	2.012616000	-0.001263000
C	-4.068882000	-1.985457000	-0.702256000
C	-4.807281000	-3.188989000	-0.614586000
C	-5.850268000	-3.322814000	0.295512000
C	-6.196723000	-2.276389000	1.151472000
C	-5.493054000	-1.059435000	1.094330000
C	-4.432180000	-0.922204000	0.164990000
H	-2.169464000	4.739889000	-0.297933000
H	2.169563000	4.739743000	-0.299317000
H	0.000043000	6.013978000	-0.432446000
H	4.530596000	-4.018494000	-1.262094000
H	6.399463000	-4.262561000	0.351146000
H	7.010649000	-2.385317000	1.868955000
H	-4.532053000	-4.016288000	-1.267075000
H	-6.400038000	-4.262331000	0.346948000
H	-7.009514000	-2.387374000	1.868232000
C	2.434666000	0.628231000	0.116943000
C	-2.434476000	0.628172000	0.116251000
N	-3.642975000	2.509103000	-0.087863000
N	3.642996000	2.508867000	-0.090820000
H	-1.644205000	-0.117203000	0.182139000
H	1.644446000	-0.117026000	0.184557000

Cl	-0.000781000	-1.737860000	-0.708084000
N	3.074021000	-1.853138000	-1.661194000
H	2.167598000	-1.454509000	-1.370082000
H	2.869994000	-2.736981000	-2.121564000
N	5.806260000	-0.020806000	1.982781000
H	5.652049000	0.908595000	1.592347000
H	6.726457000	-0.114234000	2.401555000
N	-3.075111000	-1.850399000	-1.663862000
H	-2.874168000	-2.733062000	-2.127892000
H	-2.167258000	-1.455769000	-1.371313000
N	-5.804475000	-0.023341000	1.984932000
H	-6.724498000	-0.117134000	2.404023000
H	-5.650291000	0.906605000	1.595763000

$\mathbf{2}_{G}\cdots Cl^{-}$

C	-1.216936000	4.702558000	-0.193733000
C	-1.166698000	3.298534000	-0.127704000
N	-0.000216000	2.637601000	-0.093071000
C	1.166032000	3.298967000	-0.127546000
C	1.215754000	4.703011000	-0.193565000
C	-0.000717000	5.394726000	-0.224890000
C	2.312184000	2.387499000	-0.095623000
N	3.462276000	0.561473000	-0.023454000
N	4.373811000	1.630613000	-0.079340000
C	3.874204000	-0.782301000	0.056578000
C	3.455476000	-1.774482000	-0.856587000
C	3.807548000	-3.111863000	-0.676700000
C	4.642574000	-3.486048000	0.374798000
C	5.123667000	-2.520568000	1.257189000
C	4.709200000	-1.197692000	1.107062000
N	-3.462088000	0.560288000	-0.023397000
N	-4.373943000	1.629194000	-0.079014000
C	-2.312539000	2.386662000	-0.095825000
C	-3.455094000	-1.776311000	-0.854990000
C	-3.807418000	-3.113508000	-0.674300000
C	-4.642629000	-3.486919000	0.377335000
C	-5.123560000	-2.520848000	1.259153000
C	-4.708856000	-1.198136000	1.108227000
C	-3.873809000	-0.783485000	0.057484000
H	-2.172876000	5.219717000	-0.221384000

H	2.171504000	5.220528000	-0.221091000
H	-0.000915000	6.483084000	-0.276478000
H	3.424225000	-3.835721000	-1.387692000
H	4.921320000	-4.529247000	0.503125000
H	5.786206000	-2.774894000	2.079242000
H	-3.424206000	-3.837865000	-1.384844000
H	-4.921583000	-4.529993000	0.506232000
H	-5.786158000	-2.774563000	2.081346000
C	2.189185000	1.007700000	-0.036973000
C	-2.189122000	1.006891000	-0.037301000
N	-3.650393000	2.712741000	-0.126129000
N	3.649931000	2.713955000	-0.126449000
H	-1.335334000	0.327001000	-0.004077000
H	1.335610000	0.327560000	-0.003465000
Cl	0.000487000	-1.617615000	-0.038691000
N	2.747152000	-1.451478000	-2.143202000
O	2.253564000	-2.399150000	-2.762921000
O	2.791596000	-0.282451000	-2.546088000
N	5.129020000	-0.250392000	2.178626000
O	4.256226000	0.437667000	2.714414000
O	6.328817000	-0.275890000	2.498239000
N	-2.746583000	-1.454215000	-2.141773000
O	-2.252976000	-2.402409000	-2.760711000
O	-2.790971000	-0.285486000	-2.545505000
N	-5.128408000	-0.250215000	2.179352000
O	-4.255342000	0.437615000	2.715002000
O	-6.328274000	-0.274933000	2.498728000

2$_{H}\cdots Cl^{-}$

C	-1.222864000	3.877672000	0.046923000
C	-1.170697000	2.450702000	0.027458000
N	-0.000003000	1.803256000	0.000012000
C	1.170693000	2.450699000	-0.027438000
C	1.222867000	3.877665000	-0.046912000
C	0.000003000	4.562872000	-0.000001000
C	2.355011000	1.589619000	-0.025540000
N	3.704420000	-0.118739000	-0.008307000
N	4.483955000	1.018384000	0.038278000
C	4.308058000	-1.413073000	-0.009931000
C	3.498074000	-2.557692000	0.003623000

C	4.108771000	-3.814895000	0.000848000
C	5.502135000	-3.940602000	-0.014146000
C	6.296504000	-2.787003000	-0.027106000
C	5.707759000	-1.521014000	-0.026170000
N	-3.704421000	-0.118742000	0.008275000
N	-4.483956000	1.018377000	-0.038385000
C	-2.355015000	1.589620000	0.025526000
C	-3.498075000	-2.557695000	-0.003595000
C	-4.108772000	-3.814898000	-0.000802000
C	-5.502136000	-3.940605000	0.014162000
C	-6.296506000	-2.787006000	0.027073000
C	-5.707761000	-1.521016000	0.026119000
C	-4.308059000	-1.413076000	0.009915000
H	0.000007000	5.654975000	-0.000001000
H	2.405436000	-2.490418000	0.017535000
H	3.473255000	-4.698925000	0.010750000
H	5.966439000	-4.926016000	-0.015980000
H	7.382710000	-2.869736000	-0.039696000
H	6.310280000	-0.618474000	-0.037314000
H	-2.405437000	-2.490422000	-0.017489000
H	-3.473256000	-4.698929000	-0.010669000
H	-5.966440000	-4.926019000	0.016009000
H	-7.382712000	-2.869739000	0.039638000
H	-6.310281000	-0.618477000	0.037227000
C	2.378102000	0.199964000	-0.047918000
C	-2.378104000	0.199964000	0.047913000
N	-3.658545000	2.040857000	-0.026220000
N	3.658541000	2.040862000	0.026127000
H	-1.572279000	-0.535124000	0.084585000
H	1.572276000	-0.535124000	-0.084556000
Cl	-0.000001000	-2.401488000	0.000045000
N	2.429541000	4.559006000	-0.161936000
H	3.263566000	3.994658000	0.018390000
H	2.445960000	5.505340000	0.202189000
N	-2.429529000	4.559025000	0.161975000
H	-3.263569000	3.994685000	-0.018302000
H	-2.445951000	5.505356000	-0.202162000

2$_{\mathrm{I}}\cdots Cl^-$

C	-1.166643000	3.260182000	-0.272381000

C	-1.168912000	1.845693000	-0.146674000
N	-0.000011000	1.206294000	-0.000041000
C	1.168891000	1.845682000	0.146619000
C	1.166627000	3.260166000	0.272386000
C	-0.000007000	3.965182000	0.000018000
C	2.348673000	1.000198000	0.075744000
N	3.675992000	-0.708167000	-0.071330000
N	4.438441000	0.452337000	-0.249456000
C	4.291311000	-1.996386000	-0.120630000
C	3.491613000	-3.145673000	-0.188821000
C	4.114555000	-4.395632000	-0.231485000
C	5.509385000	-4.505763000	-0.212179000
C	6.292535000	-3.346808000	-0.146996000
C	5.691223000	-2.087622000	-0.097479000
N	-3.676007000	-0.708157000	0.071344000
N	-4.438450000	0.452347000	0.249488000
C	-2.348691000	1.000210000	-0.075754000
C	-3.491627000	-3.145666000	0.188810000
C	-4.114566000	-4.395626000	0.231471000
C	-5.509396000	-4.505758000	0.212188000
C	-6.292549000	-3.346803000	0.147027000
C	-5.691240000	-2.087617000	0.097511000
C	-4.291327000	-1.996379000	0.120643000
H	-0.000005000	5.050665000	0.000031000
H	2.399777000	-3.080237000	-0.211555000
H	3.490847000	-5.286452000	-0.281572000
H	5.983773000	-5.485463000	-0.247129000
H	7.378967000	-3.419838000	-0.129150000
H	6.284866000	-1.180760000	-0.043241000
H	-2.399789000	-3.080228000	0.211517000
H	-3.490857000	-5.286446000	0.281537000
H	-5.983782000	-5.485458000	0.247137000
H	-7.378980000	-3.419835000	0.129197000
H	-6.284885000	-1.180754000	0.043286000
C	2.371817000	-0.390918000	0.122048000
C	-2.371834000	-0.390907000	-0.122064000
N	-3.625523000	1.462663000	0.157231000
N	3.625544000	1.462661000	-0.157024000
H	-1.568769000	-1.124538000	-0.245762000
H	1.568747000	-1.124550000	0.245708000

Cl	-0.000006000	-2.877166000	-0.000049000
N	2.335626000	4.036735000	0.729182000
O	2.943766000	3.613188000	1.720747000
O	2.593531000	5.093471000	0.124942000
N	-2.335627000	4.036764000	-0.729184000
O	-2.593496000	5.093524000	-0.124971000
O	-2.943581000	3.613368000	-1.720926000

$\mathbf{2}_J\cdots Cl^-$

C	1.209221000	3.931000000	-0.015149000
C	1.156351000	2.529595000	-0.021783000
N	0.000000000	1.841866000	-0.025792000
C	-1.156351000	2.529594000	-0.021793000
C	-1.209222000	3.930999000	-0.015160000
C	-0.000001000	4.647184000	-0.009966000
C	-2.356308000	1.690010000	-0.017075000
N	-3.716075000	-0.002654000	-0.008407000
N	-4.482335000	1.157725000	-0.003105000
C	-4.332262000	-1.289391000	-0.003754000
C	-3.533389000	-2.442651000	0.010634000
C	-4.156628000	-3.693444000	0.014444000
C	-5.551527000	-3.804814000	0.004676000
C	-6.334233000	-2.643573000	-0.009332000
C	-5.732950000	-1.383295000	-0.013981000
N	3.716076000	-0.002652000	-0.008390000
N	4.482336000	1.157728000	-0.003059000
C	2.356309000	1.690012000	-0.017057000
C	3.533392000	-2.442648000	0.010723000
C	4.156632000	-3.693441000	0.014518000
C	5.551530000	-3.804811000	0.004660000
C	6.334235000	-2.643570000	-0.009425000
C	5.732952000	-1.383292000	-0.014064000
C	4.332264000	-1.289389000	-0.003745000
H	2.171702000	4.439087000	-0.012984000
H	-2.171704000	4.439085000	-0.013003000
H	-2.440361000	-2.385091000	0.019010000
H	-3.530629000	-4.584180000	0.025197000
H	-6.025739000	-4.785482000	0.007809000
H	-7.421236000	-2.715625000	-0.017348000
H	-6.325986000	-0.474482000	-0.025246000

H	2.440365000	-2.385088000	0.019174000
H	3.530633000	-4.584177000	0.025329000
H	6.025743000	-4.785478000	0.007782000
H	7.421238000	-2.715622000	-0.017513000
H	6.325987000	-0.474479000	-0.025392000
C	-2.388869000	0.302946000	-0.016575000
C	2.388870000	0.302947000	-0.016574000
N	3.647945000	2.166537000	-0.008359000
N	-3.647945000	2.166535000	-0.008404000
H	1.584887000	-0.436455000	-0.019156000
H	-1.584886000	-0.436457000	-0.019130000
Cl	0.000002000	-2.286868000	0.013900000
N	-0.000001000	6.049542000	-0.057062000
H	0.843187000	6.486111000	0.304052000
H	-0.843194000	6.486111000	0.304043000

2$_K$···Cl^-

C	1.220499000	3.512191000	-0.000042000
C	1.164659000	2.104410000	-0.000014000
N	0.000000000	1.432066000	0.000039000
C	-1.164658000	2.104411000	-0.000018000
C	-1.220496000	3.512193000	-0.000054000
C	0.000001000	4.183018000	-0.000053000
C	-2.358718000	1.264093000	-0.000019000
N	-3.718555000	-0.425386000	-0.000012000
N	-4.483065000	0.739198000	-0.000048000
C	-4.339267000	-1.711238000	-0.000003000
C	-3.543180000	-2.866120000	0.000008000
C	-4.169981000	-4.115061000	0.000016000
C	-5.565213000	-4.221278000	0.000014000
C	-6.344716000	-3.057975000	0.000003000
C	-5.739913000	-1.799488000	-0.000006000
N	3.718555000	-0.425388000	-0.000001000
N	4.483066000	0.739195000	-0.000042000
C	2.358719000	1.264092000	-0.000005000
C	3.543178000	-2.866122000	0.000027000
C	4.169978000	-4.115064000	0.000024000
C	5.565210000	-4.221282000	-0.000003000
C	6.344714000	-3.057980000	-0.000030000
C	5.739912000	-1.799493000	-0.000031000

C	4.339266000	-1.711241000	0.000001000
H	2.165309000	4.042958000	-0.000052000
H	-2.165307000	4.042960000	-0.000072000
H	-2.450627000	-2.810044000	0.000013000
H	-3.547771000	-5.008372000	0.000023000
H	-6.042341000	-5.200341000	0.000022000
H	-7.431725000	-3.126845000	0.000001000
H	-6.330676000	-0.889201000	-0.000016000
H	2.450625000	-2.810045000	0.000051000
H	3.547768000	-5.008374000	0.000042000
H	6.042338000	-5.200346000	-0.000004000
H	7.431723000	-3.126850000	-0.000054000
H	6.330676000	-0.889206000	-0.000055000
C	-2.392584000	-0.123819000	-0.000009000
C	2.392584000	-0.123820000	-0.000000000
N	3.648673000	1.744773000	0.000041000
N	-3.648671000	1.744775000	-0.000021000
H	1.588739000	-0.865494000	0.000001000
H	-1.588739000	-0.865493000	0.000003000
Cl	-0.000001000	-2.650018000	0.000030000
N	0.000001000	5.679063000	0.000001000
O	-1.097839000	6.258905000	0.000121000
O	1.097844000	6.258904000	0.000131000

1_A / 2_A

C	-1.204281000	3.826288000	0.013133000
C	-1.158237000	2.420439000	0.013812000
N	-0.000000000	1.727687000	-0.000003000
C	1.158236000	2.420439000	-0.013818000
C	1.204279000	3.826288000	-0.013138000
C	-0.000001000	4.528917000	-0.000002000
C	2.391105000	1.628981000	-0.030700000
N	3.887659000	0.042798000	-0.046967000
N	4.555278000	1.257515000	-0.045973000
C	4.608652000	-1.185202000	-0.067693000
C	4.015193000	-2.337805000	-0.600010000
C	4.728527000	-3.539002000	-0.604586000
C	6.030722000	-3.590231000	-0.096353000
C	6.618627000	-2.429750000	0.420811000
C	5.913012000	-1.225542000	0.443964000

N	-3.887659000	0.042797000	0.046960000
N	-4.555279000	1.257513000	0.045966000
C	-2.391106000	1.628980000	0.030694000
C	-4.015192000	-2.337806000	0.600003000
C	-4.728525000	-3.539004000	0.604579000
C	-6.030721000	-3.590233000	0.096346000
C	-6.618627000	-2.429752000	-0.420817000
C	-5.913011000	-1.225544000	-0.443971000
C	-4.608651000	-1.185204000	0.067686000
H	-2.166257000	4.330550000	0.023553000
H	2.166255000	4.330551000	-0.023557000
H	-0.000001000	5.617515000	-0.000002000
H	3.016119000	-2.292498000	-1.025941000
H	4.267103000	-4.432998000	-1.019232000
H	6.584682000	-4.526529000	-0.106491000
H	7.631399000	-2.461581000	0.817498000
H	6.353601000	-0.319345000	0.847665000
H	-3.016118000	-2.292499000	1.025933000
H	-4.267101000	-4.432999000	1.019224000
H	-6.584680000	-4.526531000	0.106484000
H	-7.631399000	-2.461584000	-0.817503000
H	-6.353602000	-0.319347000	-0.847671000
C	2.540955000	0.252452000	-0.038479000
C	-2.540955000	0.252451000	0.038472000
N	-3.647620000	2.195702000	0.036478000
N	3.647619000	2.195703000	-0.036485000
H	-1.812408000	-0.541802000	0.010561000
H	1.812409000	-0.541802000	-0.010567000

1_B / 2_B

C	3.359718000	-0.856735000	-0.517807000
C	2.383838000	0.055421000	-0.076620000
N	1.059799000	-0.191133000	-0.169511000
C	0.663462000	-1.362465000	-0.711208000
C	1.571647000	-2.330425000	-1.177399000
C	2.936260000	-2.063484000	-1.073409000
C	-0.781934000	-1.586169000	-0.795711000
N	-2.955678000	-1.427146000	-0.711257000
N	-2.640883000	-2.654224000	-1.269751000
C	-4.312039000	-1.039048000	-0.518691000

C	-4.663723000	-0.184185000	0.533646000
C	-5.991507000	0.197708000	0.711677000
C	-7.000371000	-0.279792000	-0.148463000
C	-6.631206000	-1.150018000	-1.195059000
C	-5.304043000	-1.522683000	-1.382618000
N	2.812298000	3.322119000	1.422844000
N	4.121181000	2.917508000	1.222725000
C	2.768757000	1.339361000	0.514713000
C	1.352549000	4.763917000	2.765737000
C	1.070932000	6.003026000	3.336590000
C	1.959284000	7.086069000	3.183100000
C	3.137084000	6.884717000	2.433934000
C	3.414341000	5.651793000	1.852287000
C	2.520966000	4.584073000	2.014495000
H	4.411536000	-0.604933000	-0.417506000
H	1.195018000	-3.255530000	-1.604113000
H	3.665748000	-2.791635000	-1.424159000
H	-3.904318000	0.166086000	1.228553000
H	-6.254138000	0.863590000	1.532729000
H	-7.395063000	-1.526716000	-1.874212000
H	-5.024055000	-2.191130000	-2.191186000
H	0.672280000	3.930599000	2.924127000
H	0.159487000	6.132161000	3.918921000
H	3.834956000	7.710220000	2.299212000
H	4.321139000	5.501620000	1.274270000
C	-1.815451000	-0.747138000	-0.408331000
C	1.955228000	2.356186000	0.990604000
N	4.078764000	1.729687000	0.675750000
N	-1.335668000	-2.733595000	-1.316504000
H	0.881873000	2.456473000	1.015507000
H	-1.807386000	0.247245000	0.008776000
N	-8.323796000	0.147003000	-0.007079000
H	-8.584458000	0.488269000	0.912424000
H	-9.030137000	-0.458951000	-0.411902000
N	1.651820000	8.344882000	3.706974000
H	2.439849000	8.966471000	3.857117000
H	0.994573000	8.355609000	4.480112000

1_C / 2_C

C	-1.203906000	4.785768000	-0.000021000

C	-1.157165000	3.380029000	-0.000124000
N	-0.000001000	2.686058000	-0.000078000
C	1.157162000	3.380030000	0.000077000
C	1.203902000	4.785769000	0.000199000
C	-0.000003000	5.489038000	0.000145000
C	2.391233000	2.590442000	0.000103000
N	3.898245000	1.011155000	0.000105000
N	4.558110000	2.236979000	0.000219000
C	4.633861000	-0.200841000	0.000083000
C	3.964349000	-1.436110000	-0.000049000
C	4.694748000	-2.621171000	-0.000103000
C	6.089195000	-2.560425000	-0.000023000
C	6.767542000	-1.338717000	0.000116000
C	6.039042000	-0.153813000	0.000167000
N	-3.898246000	1.011151000	-0.000328000
N	-4.558112000	2.236975000	-0.000314000
C	-2.391236000	2.590440000	-0.000246000
C	-3.964347000	-1.436114000	-0.000378000
C	-4.694745000	-2.621175000	-0.000197000
C	-6.089192000	-2.560431000	0.000064000
C	-6.767540000	-1.338724000	0.000102000
C	-6.039042000	-0.153819000	-0.000077000
C	-4.633861000	-0.200845000	-0.000290000
H	-2.165564000	5.290529000	-0.000057000
H	2.165559000	5.290531000	0.000326000
H	-0.000003000	6.577356000	0.000237000
H	2.879772000	-1.480477000	-0.000116000
H	4.200729000	-3.587307000	-0.000213000
H	7.852711000	-1.329557000	0.000173000
H	6.539431000	0.808199000	0.000265000
H	-2.879770000	-1.480479000	-0.000546000
H	-4.200725000	-3.587311000	-0.000202000
H	-7.852709000	-1.329565000	0.000330000
H	-6.539432000	0.808192000	-0.000002000
C	2.547306000	1.217414000	-0.000032000
C	-2.547307000	1.217412000	-0.000294000
N	-3.647881000	3.164544000	-0.000287000
N	3.647878000	3.164547000	0.000240000
H	-1.813497000	0.428499000	-0.000254000
H	1.813497000	0.428501000	-0.000177000

N	6.867373000	-3.824630000	-0.000104000
O	8.105508000	-3.737947000	-0.000040000
O	6.231876000	-4.891688000	-0.000254000
N	-6.867369000	-3.824638000	0.000478000
O	-6.231871000	-4.891695000	0.000545000
O	-8.105504000	-3.737956000	0.000823000

1_D / 2_D

C	1.204192000	4.357054000	0.002826000
C	1.157438000	2.951040000	0.006649000
N	-0.001824000	2.259926000	-0.013941000
C	-1.159986000	2.952858000	-0.035868000
C	-1.204745000	4.358850000	-0.036727000
C	0.000307000	5.060287000	-0.018337000
C	-2.392394000	2.161155000	-0.061954000
N	-3.885176000	0.572069000	-0.106610000
N	-4.555570000	1.782895000	-0.071909000
C	-4.597281000	-0.663564000	-0.150127000
C	-4.001701000	-1.770960000	-0.762285000
C	-4.702447000	-2.991025000	-0.789810000
C	-5.981051000	-3.070677000	-0.216157000
C	-6.571113000	-1.944545000	0.383252000
C	-5.867685000	-0.726376000	0.423631000
N	3.879922000	0.567423000	0.094868000
N	4.551136000	1.778013000	0.066570000
C	2.388720000	2.157943000	0.038755000
C	3.980853000	-1.784268000	0.725540000
C	4.687034000	-3.001226000	0.767201000
C	5.987144000	-3.068143000	0.241482000
C	6.592007000	-1.933332000	-0.325107000
C	5.883333000	-0.719108000	-0.381515000
C	4.592728000	-0.667858000	0.146647000
H	2.166534000	4.860547000	0.018390000
H	-2.166331000	4.863770000	-0.053130000
H	0.001163000	6.149014000	-0.020247000
H	-3.027534000	-1.687029000	-1.236506000
H	-6.526022000	-4.014455000	-0.244362000
H	-6.296908000	0.158658000	0.882722000
H	2.996599000	-1.707184000	1.179429000
H	6.535082000	-4.009905000	0.278940000

H	6.320798000	0.169471000	-0.825324000
C	-2.540088000	0.784640000	-0.100805000
C	2.535202000	0.781266000	0.078190000
N	3.646384000	2.720780000	0.033562000
N	-3.649682000	2.724902000	-0.046167000
H	1.805547000	-0.012133000	0.066989000
H	-1.811685000	-0.010075000	-0.093704000
N	-4.102255000	-4.132058000	-1.342467000
H	-3.377806000	-3.945768000	-2.029292000
H	-4.749236000	-4.846616000	-1.661980000
N	-7.872456000	-2.016145000	0.889350000
H	-8.122535000	-1.295794000	1.558906000
H	-8.188419000	-2.938130000	1.171821000
N	4.123816000	-4.115353000	1.403440000
H	4.503455000	-5.014614000	1.125005000
H	3.109900000	-4.125009000	1.448596000
N	7.864941000	-2.023809000	-0.899060000
H	8.456216000	-2.767079000	-0.541146000
H	8.362999000	-1.143982000	-0.989645000

1_E / 2_E

C	-1.204004000	5.018342000	0.012068000
C	-1.156719000	3.612927000	0.013950000
N	-0.000017000	2.919061000	-0.000002000
C	1.156700000	3.612898000	-0.014183000
C	1.204014000	5.018313000	-0.012793000
C	0.000012000	5.721549000	-0.000477000
C	2.388732000	2.819943000	-0.035691000
N	3.886928000	1.237351000	-0.074344000
N	4.555268000	2.457886000	-0.041052000
C	4.615268000	0.024493000	-0.127193000
C	3.989909000	-1.154024000	-0.550897000
C	4.736187000	-2.330410000	-0.584651000
C	6.080538000	-2.381690000	-0.230214000
C	6.665796000	-1.183912000	0.175918000
C	5.968573000	0.018526000	0.242445000
N	-3.886998000	1.237453000	0.074472000
N	-4.555314000	2.457993000	0.041038000
C	-2.388769000	2.820003000	0.035592000
C	-3.990053000	-1.153795000	0.551614000

C	-4.736340000	-2.330170000	0.585578000
C	-6.080616000	-2.381548000	0.230873000
C	-6.665813000	-1.183873000	-0.175647000
C	-5.968601000	0.018562000	-0.242306000
C	-4.615355000	0.024616000	0.127532000
H	-2.165333000	5.523766000	0.021957000
H	2.165353000	5.523713000	-0.022885000
H	0.000023000	6.809781000	-0.000679000
H	2.953153000	-1.178797000	-0.866365000
H	6.644660000	-3.305986000	-0.268583000
H	6.461201000	0.926772000	0.569652000
H	-2.953352000	-1.178466000	0.867271000
H	-6.644736000	-3.305844000	0.269349000
H	-6.461181000	0.926727000	-0.569815000
C	2.537078000	1.445697000	-0.072837000
C	-2.537145000	1.445766000	0.072906000
N	-3.647755000	3.388030000	0.019856000
N	3.647731000	3.387945000	-0.020041000
H	-1.804367000	0.654718000	0.075681000
H	1.804247000	0.654693000	-0.075568000
N	4.059339000	-3.586773000	-1.036522000
O	4.742370000	-4.616246000	-1.079044000
O	2.859659000	-3.507255000	-1.338427000
N	8.112202000	-1.193288000	0.571612000
O	8.711152000	-2.273444000	0.496505000
O	8.604615000	-0.122180000	0.946787000
N	-4.059595000	-3.586400000	1.037973000
O	-2.858713000	-3.507824000	1.335308000
O	-4.741408000	-4.616852000	1.076015000
N	-8.112116000	-1.193367000	-0.571650000
O	-8.605043000	-0.121876000	-0.945032000
O	-8.711386000	-2.273248000	-0.495192000

1_F / 2_F

C	-1.204618000	4.016535000	0.018182000
C	-1.158417000	2.610794000	0.017636000
N	0.000002000	1.919280000	-0.000011000
C	1.158422000	2.610791000	-0.017667000
C	1.204627000	4.016532000	-0.018230000
C	0.000005000	4.718869000	-0.000028000

C	2.389053000	1.814722000	-0.037152000
N	3.874030000	0.214781000	-0.057894000
N	4.548525000	1.427342000	-0.094735000
C	4.575359000	-1.033326000	-0.035311000
C	4.301324000	-1.997491000	-1.030201000
C	4.957669000	-3.240042000	-0.963794000
C	5.871065000	-3.489624000	0.060399000
C	6.162287000	-2.526623000	1.025474000
C	5.520850000	-1.273604000	0.990094000
N	-3.874029000	0.214790000	0.057892000
N	-4.548522000	1.427353000	0.094720000
C	-2.389049000	1.814727000	0.037131000
C	-4.301328000	-1.997469000	1.030226000
C	-4.957677000	-3.240019000	0.963834000
C	-5.871073000	-3.489612000	-0.060356000
C	-6.162292000	-2.526622000	-1.025444000
C	-5.520852000	-1.273604000	-0.990079000
C	-4.575361000	-1.033316000	0.035324000
H	-2.166254000	4.521479000	0.032777000
H	2.166264000	4.521474000	-0.032831000
H	0.000006000	5.807545000	-0.000035000
H	4.750347000	-3.994547000	-1.720321000
H	6.377733000	-4.452392000	0.098669000
H	6.889677000	-2.729874000	1.809314000
H	-4.750357000	-3.994516000	1.720370000
H	-6.377744000	-4.452379000	-0.098615000
H	-6.889682000	-2.729881000	-1.809282000
C	2.531754000	0.435378000	-0.014576000
C	-2.531753000	0.435384000	0.014572000
N	-3.644842000	2.374393000	0.076130000
N	3.644847000	2.374384000	-0.076156000
H	-1.809002000	-0.364693000	-0.024333000
H	1.809001000	-0.364697000	0.024339000
N	3.349056000	-1.751036000	-2.025706000
H	3.201276000	-0.775645000	-2.268691000
H	3.440228000	-2.340972000	-2.846642000
N	5.757143000	-0.315932000	1.972361000
H	5.658145000	0.650581000	1.670170000
H	6.590318000	-0.475507000	2.528566000
N	-3.349061000	-1.751004000	2.025728000

H	-3.440235000	-2.340930000	2.846672000
H	-3.201278000	-0.775610000	2.268701000
N	-5.757142000	-0.315944000	-1.972358000
H	-6.590317000	-0.475524000	-2.528561000
H	-5.658142000	0.650573000	-1.670179000

1_G / 2_G

C	1.190857000	4.120711000	0.181262000
C	1.143957000	2.715444000	0.171743000
N	0.000026000	2.023322000	-0.000142000
C	-1.143940000	2.715372000	-0.172073000
C	-1.190903000	4.120636000	-0.181715000
C	-0.000039000	4.823325000	-0.000264000
C	-2.360810000	1.916851000	-0.341586000
N	-3.829383000	0.319291000	-0.497847000
N	-4.496882000	1.543194000	-0.700598000
C	-4.606175000	-0.859446000	-0.419659000
C	-4.560721000	-1.878257000	-1.388362000
C	-5.449097000	-2.953497000	-1.374081000
C	-6.416978000	-3.035929000	-0.373245000
C	-6.510153000	-2.030131000	0.589373000
C	-5.606013000	-0.970283000	0.562376000
N	3.829418000	0.319457000	0.497863000
N	4.496887000	1.543374000	0.700629000
C	2.360845000	1.916984000	0.341389000
C	4.560651000	-1.878076000	1.388451000
C	5.449010000	-2.953331000	1.374263000
C	6.416993000	-3.035782000	0.373525000
C	6.510282000	-2.029983000	-0.589085000
C	5.606156000	-0.970120000	-0.562178000
C	4.606207000	-0.859281000	0.419743000
H	2.141217000	4.626634000	0.325033000
H	-2.141290000	4.626502000	-0.325521000
H	-0.000065000	5.911728000	-0.000315000
H	-5.380233000	-3.701341000	-2.158645000
H	-7.106746000	-3.875661000	-0.351627000
H	-7.270768000	-2.052045000	1.364218000
H	5.380063000	-3.701165000	2.158831000
H	7.106759000	-3.875519000	0.351985000
H	7.270994000	-2.051894000	-1.363835000

C	-2.508242000	0.543410000	-0.262936000
C	2.508311000	0.543553000	0.262813000
N	3.595452000	2.468224000	0.613685000
N	-3.595453000	2.468057000	-0.613789000
H	1.800432000	-0.242792000	0.061351000
H	-1.800318000	-0.242918000	-0.061511000
N	-3.573017000	-1.837134000	-2.498858000
O	-2.396341000	-1.584577000	-2.206231000
O	-3.999774000	-2.088923000	-3.632389000
N	-5.707703000	0.039515000	1.657712000
O	-6.846902000	0.397730000	1.976327000
O	-4.653218000	0.394680000	2.196861000
N	3.572889000	-1.836867000	2.498893000
O	2.395736000	-1.587075000	2.205780000
O	3.999085000	-2.091361000	3.632031000
N	5.708062000	0.039773000	-1.657405000
O	4.653666000	0.395157000	-2.196582000
O	6.847320000	0.398056000	-1.975732000

1_H / 2_H

C	1.222414000	3.629804000	-0.042902000
C	1.170986000	2.196875000	-0.028041000
N	-0.000000000	1.543226000	0.000001000
C	-1.170987000	2.196875000	0.028043000
C	-1.222414000	3.629804000	0.042903000
C	-0.000000000	4.313767000	0.000001000
C	-2.368474000	1.357920000	0.040814000
N	-3.778589000	-0.314909000	0.054042000
N	-4.517821000	0.842298000	-0.004351000
C	-4.421917000	-1.585225000	0.090448000
C	-3.770033000	-2.683289000	0.667857000
C	-4.405200000	-3.927509000	0.688843000
C	-5.687670000	-4.076245000	0.150833000
C	-6.334611000	-2.969790000	-0.412717000
C	-5.707148000	-1.723608000	-0.451739000
N	3.778589000	-0.314909000	-0.054041000
N	4.517821000	0.842299000	0.004355000
C	2.368474000	1.357921000	-0.040812000
C	3.770034000	-2.683288000	-0.667860000
C	4.405201000	-3.927508000	-0.688846000

C	5.687671000	-4.076244000	-0.150835000
C	6.334611000	-2.969790000	0.412716000
C	5.707148000	-1.723608000	0.451740000
C	4.421917000	-1.585225000	-0.090447000
H	-0.000001000	5.404698000	0.000001000
H	-2.787416000	-2.562143000	1.116289000
H	-3.898330000	-4.778745000	1.139002000
H	-6.180737000	-5.045838000	0.173051000
H	-7.332487000	-3.077305000	-0.833291000
H	-6.193767000	-0.858602000	-0.891327000
H	2.787417000	-2.562141000	-1.116292000
H	3.898331000	-4.778743000	-1.139008000
H	6.180737000	-5.045837000	-0.173054000
H	7.332487000	-3.077305000	0.833291000
H	6.193766000	-0.858603000	0.891330000
C	-2.444268000	-0.027562000	0.084847000
C	2.444268000	-0.027562000	-0.084845000
N	3.663852000	1.838195000	0.010883000
N	-3.663853000	1.838195000	-0.010879000
H	1.672039000	-0.778724000	-0.101661000
H	-1.672039000	-0.778724000	0.101662000
N	-2.414795000	4.308977000	0.139116000
H	-3.274265000	3.776905000	0.001637000
H	-2.422787000	5.296572000	-0.081744000
N	2.414793000	4.308978000	-0.139115000
H	3.274264000	3.776907000	-0.001637000
H	2.422785000	5.296573000	0.081745000

1_I / 2_I

C	1.186618000	-2.984184000	-0.104545000
C	1.172338000	-1.574229000	-0.010530000
N	-0.000025000	-0.913825000	-0.000094000
C	-1.172379000	-1.574245000	0.010378000
C	-1.186642000	-2.984198000	0.104408000
C	-0.000006000	-3.697215000	-0.000060000
C	-2.375323000	-0.762462000	-0.130361000
N	-3.838787000	0.846272000	-0.165924000
N	-4.425943000	-0.283734000	-0.725418000
C	-4.569686000	2.064936000	-0.048972000
C	-3.891937000	3.290882000	-0.057781000

C	-4.618996000	4.476723000	0.070651000
C	-6.012266000	4.441212000	0.191049000
C	-6.679166000	3.210533000	0.184800000
C	-5.964664000	2.016686000	0.070761000
N	3.838735000	0.846303000	0.165901000
N	4.425839000	-0.283683000	0.725489000
C	2.375274000	-0.762434000	0.130256000
C	3.891930000	3.290912000	0.057695000
C	4.619022000	4.476736000	-0.070698000
C	6.012302000	4.441198000	-0.190973000
C	6.679180000	3.210507000	-0.184640000
C	5.964647000	2.016676000	-0.070637000
C	4.569658000	2.064954000	0.048974000
H	0.000003000	-4.783219000	-0.000037000
H	-2.812588000	3.320447000	-0.185088000
H	-4.094155000	5.429741000	0.063330000
H	-6.575201000	5.367268000	0.285757000
H	-7.762581000	3.176383000	0.278104000
H	-6.466875000	1.054256000	0.072648000
H	2.812570000	3.320498000	0.184907000
H	4.094198000	5.429764000	-0.063444000
H	6.575263000	5.367241000	-0.285650000
H	7.762602000	3.176335000	-0.277849000
H	6.466839000	1.054237000	-0.072458000
C	-2.559459000	0.570418000	0.200012000
C	2.559440000	0.570436000	-0.200146000
N	3.539801000	-1.235482000	0.692203000
N	-3.539902000	-1.235533000	-0.692182000
H	1.909567000	1.282515000	-0.684219000
H	-1.909542000	1.282511000	0.684005000
N	-2.424871000	-3.755277000	0.378668000
O	-3.133740000	-3.354376000	1.309621000
O	-2.620661000	-4.768393000	-0.304783000
N	2.424907000	-3.755225000	-0.378635000
O	2.620757000	-4.768186000	0.305030000
O	3.133868000	-3.354355000	-1.309530000

1_J / 2_J

C	-1.201292000	3.564712000	0.035579000
C	-1.150334000	2.166830000	0.030516000

N	0.001038000	1.458738000	0.014057000
C	1.153569000	2.165058000	0.002387000
C	1.206805000	3.562843000	0.008350000
C	0.003352000	4.290570000	0.024812000
C	2.392034000	1.379787000	-0.021125000
N	3.891438000	-0.204199000	-0.042279000
N	4.557231000	1.010930000	-0.052196000
C	4.613390000	-1.431012000	-0.062995000
C	4.012457000	-2.590280000	-0.572331000
C	4.727168000	-3.790691000	-0.577048000
C	6.038270000	-3.835497000	-0.091705000
C	6.633494000	-2.668837000	0.402769000
C	5.926833000	-1.465274000	0.425884000
N	-3.892093000	-0.198264000	0.052525000
N	-4.556146000	1.017832000	0.043392000
C	-2.390178000	1.383452000	0.041416000
C	-4.024083000	-2.579889000	0.599686000
C	-4.740614000	-3.779219000	0.603972000
C	-6.044888000	-3.826059000	0.100758000
C	-6.631413000	-2.662536000	-0.411198000
C	-5.922679000	-1.460185000	-0.433994000
C	-4.616093000	-1.423883000	0.072784000
H	-2.166348000	4.065666000	0.050322000
H	2.172706000	4.062334000	0.001233000
H	3.005585000	-2.551073000	-0.979983000
H	4.259208000	-4.689515000	-0.973639000
H	6.593102000	-4.771305000	-0.101697000
H	7.653143000	-2.695316000	0.781985000
H	6.373198000	-0.554456000	0.812407000
H	-3.023041000	-2.538755000	1.021257000
H	-4.279648000	-4.675460000	1.014353000
H	-6.601254000	-4.760958000	0.110572000
H	-7.645794000	-2.690574000	-0.804185000
H	-6.362355000	-0.551793000	-0.833666000
C	2.543845000	0.004152000	-0.024256000
C	-2.544072000	0.008096000	0.052193000
N	-3.645065000	1.953584000	0.037260000
N	3.647632000	1.948062000	-0.039561000
H	-1.816143000	-0.786800000	0.031761000
H	1.815231000	-0.789511000	0.012866000

N	0.005131000	5.674385000	0.082130000
H	-0.850178000	6.151633000	-0.178606000
H	0.854604000	6.150305000	-0.199291000

1_K / 2_K

C	-1.214026000	3.143152000	0.011292000
C	-1.160734000	1.736611000	0.011767000
N	0.000003000	1.048537000	0.000015000
C	1.160738000	1.736614000	-0.011722000
C	1.214027000	3.143156000	-0.011186000
C	-0.000001000	3.817033000	0.000065000
C	2.394067000	0.950233000	-0.026485000
N	3.897620000	-0.626420000	-0.043060000
N	4.558013000	0.593719000	-0.034716000
C	4.628010000	-1.850219000	-0.065570000
C	4.050333000	-3.000031000	-0.620094000
C	4.772021000	-4.196137000	-0.626230000
C	6.066191000	-4.243414000	-0.097402000
C	6.638068000	-3.085014000	0.441710000
C	5.923957000	-1.885834000	0.466456000
N	-3.897615000	-0.626427000	0.042944000
N	-4.558010000	0.593710000	0.034433000
C	-2.394063000	0.950227000	0.026422000
C	-4.050388000	-2.999979000	0.620205000
C	-4.772077000	-4.196085000	0.626389000
C	-6.066194000	-4.243414000	0.097437000
C	-6.638014000	-3.085069000	-0.441855000
C	-5.923899000	-1.885893000	-0.466653000
C	-4.628008000	-1.850224000	0.065505000
H	-2.161026000	3.670385000	0.020129000
H	2.161024000	3.670393000	-0.020054000
H	3.058301000	-2.956497000	-1.062555000
H	4.324106000	-5.088573000	-1.058453000
H	6.626863000	-5.175629000	-0.109106000
H	7.644581000	-3.114309000	0.853954000
H	6.351996000	-0.981162000	0.886822000
H	-3.058399000	-2.956402000	1.062759000
H	-4.324205000	-5.088477000	1.058747000
H	-6.626869000	-5.175626000	0.109181000
H	-7.644484000	-3.114406000	-0.854200000

H	-6.351893000	-0.981264000	-0.887159000
C	2.550983000	-0.426167000	-0.038398000
C	-2.550978000	-0.426173000	0.038402000
N	-3.645630000	1.525533000	0.025096000
N	3.645633000	1.525541000	-0.025345000
H	-1.827895000	-1.225741000	0.015952000
H	1.827904000	-1.225735000	-0.015839000
N	-0.000001000	5.318644000	0.000078000
O	1.099349000	5.887625000	-0.013816000
O	-1.099356000	5.887621000	0.014100000
